# Model of ligand-triggered information transmission in G-protein coupled receptor complexes

**DOI:** 10.3389/fendo.2023.1111594

**Published:** 2023-05-09

**Authors:** Roger D. Jones, Alan M. Jones

**Affiliations:** ^1^ Department of Biology, University of North Carolina at Chapel Hill, Chapel Hill, NC, United States; ^2^ European Centre for Living Technology, Ca’ Foscari University of Venice, Venice, Italy; ^3^ Systems Engineering and Research Center, Stevens Institute of Technology, Hoboken, NJ, United States; ^4^ Department of Pharmacology, University of North Carolina at Chapel Hill, Chapel Hill, NC, United States

**Keywords:** G protein coupled receptor, drug discovery, information transmission, maximum rate of entropy production, transmembrane receptor, barcode, flute model, QR code

## Abstract

We present a model for the effects of ligands on information transmission in G-Protein Coupled Receptor (GPCR) complexes. The model is built *ab initio* entirely on principles of statistical mechanics and tenets of information transmission theory and was validated in part using agonist-induced effector activity and signaling bias for the angiotensin- and adrenergic-mediated signaling pathways, with *in vitro* observations of phosphorylation sites on the C tail of the GPCR complex, and single-cell information-transmission experiments. The model extends traditional kinetic models that form the basis for many existing models of GPCR signaling. It is based on maximizing the rates of entropy production and information transmission through the GPCR complex. The model predicts that (1) phosphatase-catalyzed reactions, as opposed to kinase-catalyzed reactions, on the C-tail and internal loops of the GPCR are responsible for controlling the signaling activity, (2) signaling favors the statistical balance of the number of switches in the ON state and the number in the OFF state, and (3) biased-signaling response depends discontinuously on ligand concentration.

## 1 Introduction

This study examines the consequences and implications of a novel thought experiment that posits that natural selection promotes the design of molecular information-processing systems that maximize the efficiency of information processing and maximize the rate of entropy production. The implications of the thought experiment are compared with existing experimental observations that either support or are consistent with the conclusions of the thought experiment [Rajagopal et al. (1); Zielińska and Katanaev (2); Keshelava et al. (3–5). The conclusions suggest a novel approach to drug discovery and targeting that depends on an enhanced importance of dephosphorylation over phosphorylation, on switch interactions that tend to maintain a balance between phosphorylated and unphosphorylated sites, and on discontinuous response to continuous increases of free-ligand concentration. The study starts with a very general treatment of molecular systems from a statistical-mechanics point of view and then specializes to the structural and physiological details of specific transmembrane information-processing systems.

The approach here complements more traditional modeling approaches (6), such as that described in in the Operational model of Black and Leff (7, 8) and ternary complex models (TCM) [Weiss et al. (9); Onaran et al. (10)], which focus on the ternary reaction in the receptor complex. Unlike earlier models, the approach here accounts for protein flexibility and rigidity. The picture that emerges is one of flexible protein matrix embedded with long-lived rigid bonds. This allows for alteration of protein conformations among multiple proteins at once. The model developed here permits the maximization of information transmission efficiency [Shannon and Weaver (11)] and the efficiency of optimization of free energy and entropy [Jaynes (12)]. We dub these two principles as, respectively, Maximum Information Storage and Transmission (MIST), commonly known as maximum capacity, and Maximum Rate of Entropy Production (MREP), commonly known as Maximum Entropy Production. The thought experiment begins at a high level and then specializes.

We begin at the level of statistical mechanics and natural selection. To respond to their environment, living cells sense conditions external to the cell, transmit that information across the cell membrane, and generate chemical reactions that alter the structure and behavior of the organism to the organism’s evolutionary advantage [Lefkowitz (13)]. External stimuli may be either chemical ligands, such as nutrients [Urano and Jones (14)] and hormones [Ahlquist (15)], or physical such as in the form of photons [Rieke and Baylor (16)] and forces [Storch et al. (17)]. We focus on ligand stimuli here. The initial high-level version of the thought experiment imagines an ensemble of binary molecular switches located at fixed points in space. Given levels of entropy and information flow through the ensemble and the individual switches. The flexible parts of proteins respond to the flow by changing conformations to conformations that optimize the flow.

The high-level thought experiment is then specialized to address particular conditions within a biological context. Information processing and transmission at the molecular level, in many systems, is performed by a set of complexes that includes a receptor protein that traverses the cell membrane and transmits stimulus information from the extracellular to intracellular environment and a set of transducer proteins that carry the stimulus signal to intracellular sites that effect a response to the external stimulus. Extracellular stimuli that activate transmembrane signals may trigger multiple and varied responses to stimuli. In animals, for instance, a portion of the spectrum of responses can be encoded in independent molecular structures that collectively behave like a common barcode [Tobin et al. (18); Tobin (19); Yang et al. (20); Chakravorty and Assmann (21); Latorraca et al. (5); Chen et al. (22); Tunc-Ozdemir et al. (23)]. Studies have addressed the quantitative flow of information through signaling complexes [Keshelava et al. (3); Selimkhanov et al. (24)]. The observations indicate that the complexes are only seen transmitting one or, at most, about two bits of information from a stimulus ligand, yet the number of effector couplings observed require much more than that amount of information in order to trigger the couplings [Rajagopal et al. (1); Latorraca et al. (5)]. This provides the context for the specialized thought experiment.

**Figure 1:**
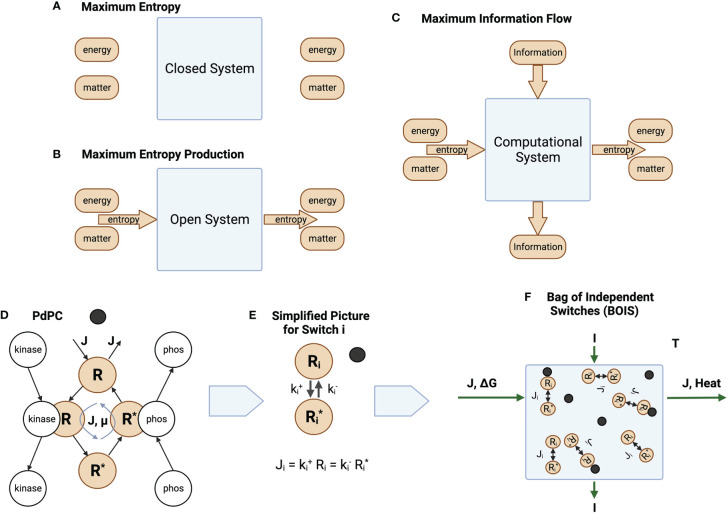
Information and Entropy Flow. **(A)** An isolated closed system is one in which no energy, matter, or entropy enters or leaves the system. **(B)** An open ystem is one in which matter can enter and leave the system. Energy may also enter and leave the system if the system is not isolated. This generates a flow of entropy. **(C)** Information may also enter and leave the system. **(D)** The phosphorylation and dephosphorylation of a receptor. The switch becomes ACTIVE after a ligand (black circle) binds to a receptor. It is INACTIVE before the ligand binds. For ACTIVE switches, a chemical flux J of receptor flows through the states $R\Leftrightarrow R^{*}$ . The matter and energy fluxes are driven by a sources external to the switch cycle. For ACTIVE switches, the unphosphorylated (OFF) state is given by a high concentration of the receptor in state R , while the phosphorylated (ON) state is given by a high concentration of the receptor in state $R^{*}$ . A receptor dissipates an amount of heat −μ as flux completes a round trip through the switch states. **(E)** Simplified notation. We reduce the picture in D to its essential elements, the OFF/ON states and the rates $k^{+}$ and $k^{-}$ at which the states transition to each other. Whether a switch is ON or OFF is determined by the values of the rates. A low value of $k_{i}^{-}$ forces the switch to turn ON, while a high value forces the switch OFF. **(F)** A collection of switches coupled by a common flux source J , a common free-energy source ΔG , and information flow I . The same amount of matter that enters the system leaves the system. The free energy is converted to heat that is absorbed and dissipated by an enclosing heat bath at temperature T .

The characterization of the general thought experiment is described in **Figure 1**. In a closed and isolated chemical system, **Figure 1A**, one in which no matter or energy enters or leaves the system, entropy is maximized [Reif (25)]. Since the entropy is maximized, the rate of production of entropy at the maximum is zero.

Living biological systems are, however, not closed, isolated systems. Matter and the internal energy contained in matter flow through organisms. They are open systems. This is illustrated schematically in **Figure 1B**, which illustrates an open system in which matter is forced through the system in the form of chemical flux. Living biological systems fall into this category. Degraded energy is released as heat. Sometimes a portion of the energy might be released in the form of useful work. This is the case, for instance, if the open system is a muscle.

Unlike the isolated systems of **Figure 1A**, open biological systems do not achieve a state of maximum entropy. Entropy in the form of a net chemical and energy fluxes continuously flows through the system preventing the system from reaching equilibrium. One might speculate that the system organizes itself such that the system moves in the direction of equilibrium as quickly as possible given the constraints on the system. This is the principle of MREP [Jaynes (12)]. It should be emphasized that MREP is not the Second Law of Thermodynamics [(26, Chapter 3)] in which entropy itself is maximized. MREP maximizes the rate of entropy production, not entropy itself.

Biological processes also adapt to their environment. This is illustrated in **Figure 1C** where an additional flow is in the form of information from the environment to output responses. Molecular information processes are usually mediated by chemical switches. The phosphorylation-dephosphorylation cycle (PdPC) and the GTPase cycle (GTPC) are the two most common switches [Qian (27)]. The switches are triggered by a stimulus, often a ligand ([3]), that turns a switch from OFF to ON. The configuration of OFF/ON switches trigger particular downstream responses [Latorraca et al. (5)].

One requirement for adaptability is that biological processes must be repeatable (28). A cell or organism must be able to repeat a response process every time it encounters a given environmental trigger. This implies that processes exist as cycles, whether they are metabolic cycles such as Krebs cycle or they are systems of switches. Every cycle must eventually return to its original state, which may be any of the states in a cycle. This puts severe constraints on the mechanisms by which information flows in a process.

While the model is valid for both PdPC and GTPC switches, we will tend to use PdPCs as our exemplar for definiteness and simplicity. **Figure 1D** illustrates a PdPC. Here, a ligand (dark circle) binds to a receptor *R*. A chemical flux J through the receptor states is initiated and an amount of free energy μ is dissipated as heat by each receptor transiting the cycle. Switches that do not maintain a chemical flux are denoted as INACTIVE, while switches that maintain a chemical flux are ACTIVE. The receptor R of an ACTIVE switch is then phosphorylated to state $R^{*}$ with the aid of a catalyst kinase and dephosphorylated with the aid of a catalyst phosphatase. Information theory deals with switches that are completely ON or completely OFF. The receptor can be in many different conformational states. Switches are composed of receptor states. The switch is ON if the probability of finding a receptor in state $R^{*}$ is large. The switch is OFF otherwise.

We can simplify the picture in **Figure 1D** by absorbing the catalytic activity into the reaction rates $k_{i}^{+}$ and $k_{i}^{-}$ for the forward and return rates for switch i , respectively. The chemical flux for switch *i* is illustrated in **Figure 1E**.


(1) $$J_{i}=k_{i}^{+}R_{i}=k_{i}^{-}R_{i}^{*}$$

We assume that many computational processes can be reduced to an ensemble, or bag, of independent switches (BOIS) illustrated in **Figure 1F**. Here, we have a number of switches that are located in space. They may be associated with different positions on a transmembrane receptor, for instance. Each switch is associated with one location. The configuration of the population of switches is determined by whether a switch is ON or OFF at each location. The ON/OFF pattern of switches contains the information that can be transmitted to downstream processes. The ensemble is assumed to transmit both information and matter/energy. This BOIS model is significantly simpler that most detailed biological-process models and may be the simplest molecular-information model that is amenable to techniques of statistical physics.

The systems illustrated in **Figures 1B, C** are very general and scalable. Every living biological process, whether it is metabolic as in the Krebs cycle, information-bearing as in biochemical switches [Qian and Reluga (29); Qian (27)], or behavior of a complete cellular organism, can be mapped to this general picture.

We focus here on the BOIS model shown in **Figure 1F**. The two basic optimization principles, MIST and MREP, can be stated explicitly.

### 1.1 Maximum information storage and transmission

Information storage and transmission is maximized in molecular computation processes.

Information and entropy are related concepts. In fact, entropy and information can be transformed into each other [Szilard (30)]. In biological systems, which are also physical systems, entropy and information are fungible. This leads us to a principle for entropy similar to our principle for information. We use the principle of MREP (Jaynes (12); Martyushev and Seleznev (31–33) as the second general principle for biological information processing.

### 1.2 Maximum entropy production

In an open, far-from-equilibrium (nonequilibirum) steady state (NESS), the observed conformation of the steady state is the one that produces the maximum rate of entropy production given externally applied constraints of chemical, free-energy, and information fluxes [Jaynes (12)].

The maximization of entropy and information flux in the complete system of switches requires the flux to be variable at the switch level. The concentrations and fluxes within each switch adapt to maximize the total information and entropy fluxes. For this to occur, the switches must be flexible [Engh and Bossemeyer (34); Karshikoff et al. (35); Gerstein and Echols (36); Marsh et al. (37); Jacobs et al. (38); Sauer et al. (39); Bernadó et al. (40); Teilum et al. (41); Homans (42); Teilum et al. (43)]. The flexibility of proteins within the switches implies that reaction rates among protein conformations can be variable. Storage of molecular information requires that not all conformation states are flexible, however. Some reaction rates must be rigid for information storage to be stable. This switch ensemble has been described by Schrödinger as an aperiodic crystal [Schrodinger et al. (44)].

The sequestering of reactions into flexible and rigid categories is determined by the background free energy. In systems near equilibrium, the background energy is supplied by thermal fluctuations at temperature T . If free energy is injected into the system by an external process such as phosphorylation, then the random fluctuations in the neighborhood of the injection may be much higher than thermal fluctuations. The free-energy injected by an ATP is about 12 kcal/mol [(45, Sec. 15.2) Qian and Reluga (29)], which is to be compared with 0.6 kcal/mol for thermal fluctuations. Reactions with free-energy barriers smaller than the fluctuation energy are flexible, while reactions with barriers larger than the fluctuation energy are rigid.

It should be noted that MREP is consistent with the Second Law of Thermodynamics. The Second Law describes the final equilibrium state of a system. MREP describes how quickly that equilibrium state is approached.

## 2 The basic mathematical model

### 2.1 Maximum information storage and transmission

An ensemble of switches is a collection of M switches, $R_{T}$ receptors, and L ligands in which the switches present ON and OFF states. Macroscopic quantities are calculated by taking an average of switch properties over the entire ensemble. An equivalent approach is the time-average picture in which a single switch or group of switches experiences all possible allowable states over time. Macroscopic quantities are calculated by taking a time-average of the switch trajectories through their state space. The two approaches are equivalent according to the Ergodic Hypothesis [Reif (25)]. Both pictures are useful for our purposes

An ACTIVE switch maintains a finite chemical flux [Qian (46)]. Consider first an ensemble of M ACTIVE switches that support a chemical flux $J_{i}$ for each switch i as illustrated in **Figure 1F**. Here, INACTIVE switches are defined as those switches that do not support a chemical flux. The number of ACTIVE switches in the ON state is $M_{o}$ , while the number in the OFF state is $M-M_{o}$ . A given configuration $x_{w}\in X\left(M,M_{o}\right)$ of $M_{o}$ ON states among M ACTIVE switches is, after Boltzmann, called a *complexion* [CERCIGNANI (47)]. As an example, if the ON state is designated by one and the OFF state by zero, then the set of complexions for M=4 switches with $M_{0}=2$ of the switches being ON is (1 1 0 0), (1 0 0 1), (1 0 1 0), (0 0 1 1), (0 1 1 0), and (0 1 0 1), for a total of six complexions. We assumed the switches are distinguishable. Each switch can be found in a well-defined location.

The amount of information H(X) in a given ensemble of switches is an average of the logarithm of the probability $\mathrm{Pr}\;(x_{i})$ of finding the switch in a given complexion [Shannon and Weaver (11)].


(2) $$H\left(X\right)=-\sum_{w=1}^{W}\mathrm{Pr}(x_{w})\log(\mathrm{Pr}(x_{w}))$$

where $\mathrm{Pr}(x_{w})$ is the probability of the ensemble of switches being found in complexion $x_{w}$ . If all the switches in a probability distribution have the same probability 1/W , then then Eq. 2 becomes


(3) H(X)=log W

The number of configurations of M switches with $M_{o}$ in the ON state is


(4) $$W\begin{array}{ll} =\frac{M!}{M_{o}!\left(M-M_{o}\right)!}\leq \frac{M!}{\left(M/2\right)!\left(M/2\right)!} & \;\;\;\;\;\left(M\text{ even}\right) \end{array}$$

We see from Eq. 4 that the maximum information storage in Eq. 3 occurs, for a given switch number M and a given number of ON switches $M_{o}$ , when the number of ON switches in a spectrum of M ACTIVE switches is half the total number of ACTIVE switches M .


(5) $$\begin{array}{ll} M_{o}=M-M_{o}=\frac{M}{2} & \;\;(M\text{ even}) \end{array}$$

If we assume that natural selection favors systems that maintain maximum information storage in a collection of switches, then this implies that a switch configuration that has equal numbers of ON and OFF switches tends to be favored. This is the justification for prediction (2) outlined in the abstract.

**Figure 2:**
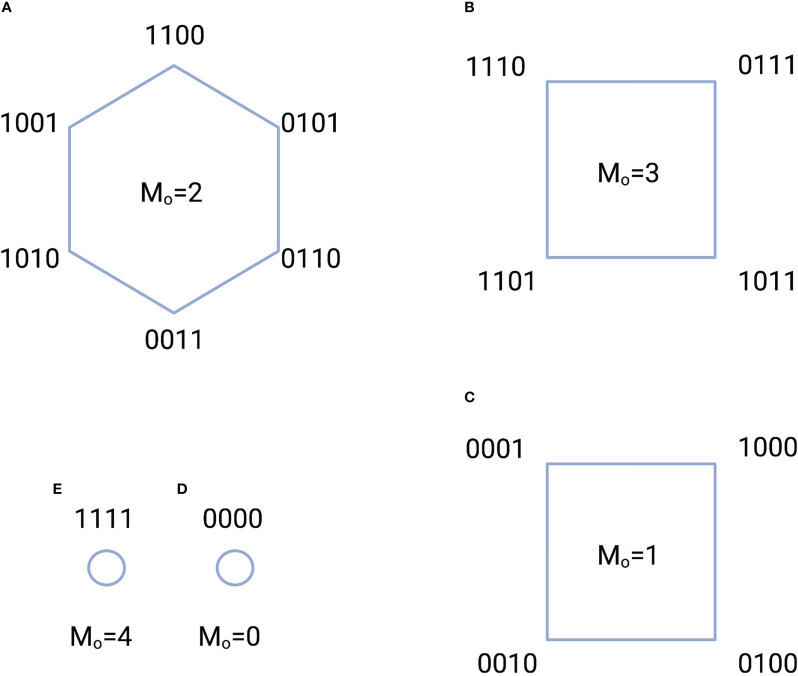
Five configuration groups **(A-E)** characterized by the total number of ACTIVE switches M=4 and the number of ON states $M_{o}=0,1,2,3,4$ . The total number of switch configurations, or complexions, is 16. A “1” indicates an ON state, while a “0” indicates an OFF state. For example, the notation 1100 indicates that the switch in location 1 is ON; the switch in location 2 is ON, while the switches in locations 3 and 4 are OFF. The amount of information stored in each group is equal to the logarithm of the total number of switches in the group. For instance, the information in the group of A is log (6)=2.58 bits.

We take as an example the configurations of switches that contain M=4 ACTIVE switches. These configurations are displayed in **Figure 2** where the switches have been assembled into five groups based the number of switches that are ON. The information stored in each group is the logarithm of the number of switches in the group. Here, the group in **Figure 2A** has the highest information content.

Shannon and Weaver (11) defined the information I(Y|X) transmitted from one distribution Pr(X) to another distribution Pr (Y) as


(6) I(Y|X)=H(Y)−H(Y|X)=H(X)−H(X|Y)

where H(Y|X) is the information stored in the conditional probability Pr(Y|X) . Here, X might be the switch ON/OFF spectrum and Y might be the spectrum of effector coupling, whether a given effector is activated or not. We see that maximum information transmission occurs when the storage terms H(X) and H(Y) are maximal and the conditional terms *H*(*X* | *Y*) and H(Y|X) are minimal. The conditional terms are zero if the mapping from X to Y and Y to X is unique in each direction. Every single state in X maps to a single state in Y and every single state in Y maps to a single state in X . Intuitively, the transition from X to Y is like a deck of cards that is shuffled. The amount of information stored in the cards is the same before and after shuffling, but the order of the cards is different. The maximum information transmission that is possible in a process is known as the capacity $C_{I}$ of the system [Shannon and Weaver (11)].

### 2.2 Constancy of receptor concentration in switches

We can maximize the information stored in the receptor concentrations of each switch in **Figure 1F**. The total receptor concentration $R_{T}$ is equal to the receptor concentration $R_{S}$ in the switches plus the concentration ΔR of any receptors not attached to a switch.


(7) $$R_{T}=R_{S}+\text{Δ}R$$

The probability Pr(i|j) that receptor j that is associated with any switch is associated with switch *i*


(8) $$\mathrm{Pr}\;(i|j)=\frac{R_{i}+R_{i}^{*}}{R_{S}}=\frac{R_{i}+R_{i}^{*}}{R_{T}-\Delta R}$$

where $R_{i}+R_{i}^{*}$ is the number of receptors, both ON and OFF, associated with switch i . A uniform distribution maximizes information storage.


(9) $$\mathrm{Pr}\;(i|j)\to \frac{1}{M_{S}}$$

which yields


(10) $$R_{i}+R_{i}^{*}=\frac{R_{T}-\Delta R}{M_{S}}=R_{ref}$$

  where $R_{ref}$ is a reference concentration that is independent of the number of ACTIVE switches, the number of ON switches, and the number of OFF switches.

The constant concentration defined in Eq. 10 is a convenient set of units to measure receptor concentration. If the switch is ON, then


(11) $$\frac{R_{i}^{*}}{R_{ref}}\to 1$$

This normalization is experimentally accessible and is currently used in assay experiments to supply a set of units for receptor concentration. It is observed in assay experiments that the maximum response for many ligands and downstream coupling pathways have similar values [Rajagopal et al. (1)]. The maximum response to a particular ligand in this group is taken to be the reference receptor concentration $R_{ref}$ . Here, we normalize all receptor concentrations to $R_{ref}$ .

### 2.3 Maximum rate of entropy production

Proteins can have both flexible and rigid domains [Engh and Bossemeyer (34); Karshikoff et al. (35); Gerstein and Echols (36); Marsh et al. (37); Jacobs et al. (38); Sauer et al. (39); Bernadó et al. (40); Teilum et al. (43); Homans (42); Teilum et al. (41)]. The principle of MREP states that flexible internal degrees of freedom within the NESSs adjust such the naturally preferred NESS generates entropy at a maximum rate, consistent with relevant constraints such as mass and energy conservation [Jaynes (12); Dobovišek et al. (33)]. The maximum is not only the maximum rate over all possible NESSs, but also over all flexible internal states of each NESS. The receptors are flexible for bonds with binding energies below some energy determined by the distance from equilibrium and rigid for bonds with greater binding energies [Dobovišek et al. (33)].

Note that the MREP picture is quite different from a traditional mass-action treatments, such as the Operational model and TCM. [Black and Leff (7); Stephenson (8); De Lean et al. (48); Onaran et al. (10)]. The key difference results from protein flexibility and rigidity. In a mass-action simulation, the concentrations are allowed to vary, while reaction rates are held constant. The mass-action approach assumes that all reaction rates are rigid. For MREP, only rigid rates are held constant, while concentrations and flexible rates vary. This implies that fluxes $J_{i}$ and free-energy drops $\mu_{i}\leq 0$ can vary for flexible reactions.

In anticipation of specializing the results to a GPCR complex, we add a GTPC to our collection of PdPCs. We consider the simple case in which we have two types of ACTIVE switches, N PdPC switches and one GTPC. Here, M=N+1 . The collection of ACTIVE PdPCs obey a chemical reaction of the form


(12) $$R_{i}\overset{k_{i}+}{\underset{k_{i}-}{⇌}}R_{i}*$$

where $R_{i}$ is the receptor state in which the switch i is unphosphorylated (OFF) and $R_{i}^{*}$ is the phosphorylated (ON) state. Here, $k_{i}^{-}$ is the reaction rate from the ON state to the OFF state and $k_{i}^{+}$ is the rate from the OFF to the ON. It is the rate at which the ON state is dephosphorylated.

The GTPC is of the form


(13) $$R_{G}\overset{k_{G}+}{\underset{k_{G}-}{⇌}}R_{G}^{*}+G$$

Here, $R_{G}$ is the receptor concentration associated with the OFF state and $R_{G}^{*}$ is the receptor concentration associated with the ON state of the GTPC, and G is the $G_{\alpha }$ bound to GTP that effects the downstream effector coupling. Here, $k_{G}^{+}$ and $k_{G}^{-}$ are the forward and backward reaction rates, respectively. We assume the number of $G_{\alpha }GTP$ is much larger than the number of free receptors.

Consider a single given receptor in a given volume V that can be in a number of internal states. The flow among the states is determined by the probability of the receptor being in a certain state and the reaction rates among the states. The total receptor concentration is 1/V receptors per unit volume. The entropy production rate σ for all the ACTIVE switches in the receptor is


(14) $$T\sigma =-\sum_{i=1}^{N}\;J_{i}\mu_{i}-J_{G}\mu_{G}$$

(15) $$J_{i}=\mathrm{Pr}\;(O\mathrm{N|}i)k_{i}^{-}$$

(16) $$J_{G}=\mathrm{Pr}\;(O\mathrm{N|}G)k_{G}^{-}$$

(17) Pr(OFF|i)=1−Pr(ON|i)

(18) Pr (OFF|G)=1−Pr(ON|G)

where T is the temperature of the heat bath in energy units and $\mu_{i}\leq 0$ is the free-energy cost for turning ACTIVE switch i to ON and then back to OFF with similar conditions for the GTPC. Here, Pr(ON | i) is the probability that ACTIVE switch i is ON, and Pr(OFF | i) is the probability that the switch is OFF. Here, $J_{i}$ is the flux per receptor. The summation is over N , the total number of PdPCs.

Since, from Eq. 10, the number of receptors in each switch is constant, we can express the chemical flux $J_{i}$ in terms of the probability a switch is ON or OFF, Eqs. 15 and 16. The ON-state receptor concentration is


(19) $$R_{i}^{*}=\mathrm{Pr}(O\mathrm{N|}i)R_{ref}$$

with comparable expressions for the GTPC and the OFF states.

The total free-energy drop ΔG is finite. We quantify this by setting the average drop $\mu_{0}$ over switches to be constant.


(20) $$M\mu_{0}=\sum_{i=1}^{N}\mu_{i}+\mu_{G}=\Delta G$$

The entropy production, Eq. 14, along with normalization and free-energy constraints can be maximized with the method of Lagrange multipliers (see **Supplement**). We find that the chemical flux is constant $J_{0}$ in every switch


(21) $$J_{i}=J_{G}=J_{0}=\frac{J}{M}$$

where J is the total chemical flux into the system.

Equation 21 can be used to identify the control point for turning a switch ON and OFF. From the definition of flux, Eqs. 15 and 16, the probability of a PdPC being ON is


(22) $$\mathrm{Pr}(ON|i)=\frac{J_{0}}{k_{i}^{-}}$$

and the probability of the GTPC being ON is


(23) $$\mathrm{Pr}(ON|G)=\frac{J_{0}}{k_{G}^{-}}$$

As can be seen from Eqs. 22 and 23, the ON states of the switches are completely determined by the externally applied chemical flux and the return rates $k_{i}^{-}$ and $k_{G}^{-}$ for the switches. These two reaction rates are the dephosphorylation rate in PdPCs and the backward rate for the reaction in Eq. 13 for the GTPC. This is prediction (1) in the abstract. The switch is ON when the reaction rate, $k_{i}^{-}$ or $k_{G}^{-}$ , is equal to the flux $J_{0}$ . The switch is OFF when the rate is much larger than $J_{0}$ . In the PdPCs, this rate is the rate catalyzed by the phosphatase. In the GTPC, this is the hydrolysis/association rate for GTP-bound G protein to bind to the receptor. To maintain a stable switch, the return rates $k_{i}^{-}$ and $k_{G}^{-}$ must be rigid, not flexible. This indicates that the return rates are the controls for turning the switches ON and OFF.

### 2.4 Specialization of the model to the GPCR complex

**Figure 3:**
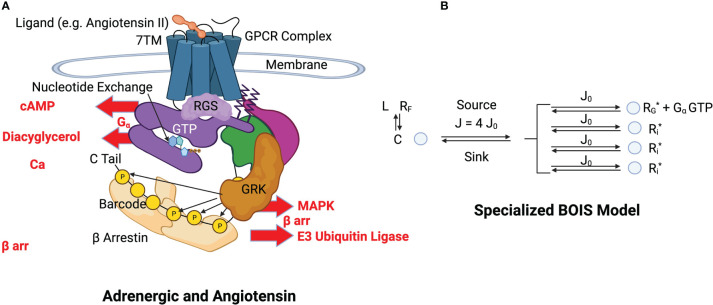
**(A)** Structural schematics of adrenergic and angiotensin receptors. A ligand, indicated in orange at the top of the figure, binds to the transmembrane receptor causing a conformational change in the GPCR complex [Lefkowitz (13)] (see this reference for details and definitions). The GPCR simultaneously binds to a GDP-bound heterotrimeric G-protein complex (purple, green and magenta). The bound GDP is converted to GTP by nucleotide exchange. Intrinsic GTP hydrolysis by the $G_{\alpha }$ subunit is accelerated by a Regulator of G-Protein Signaling (RGS). The $G_{\alpha }$ subunit (purple) and the β - γ subunits (green and magenta) of the G protein separate. The GTP-bound $G_{\alpha }$ complex is released from the GPCR and activates downstream $G_{\alpha }$ processes such as those indicated by cAMP, Diacyglycerol, and Ca. This constitutes a GTPC. Phosphorylation sites are selectively phosphorylated with a kinase and dephosphorylated by a phosphatase. The configuration of OFF and ON states in the barcode determine the activation and inactivation of β arr downstream responses such as those indicated by MAPK and E3. **(B)** BOIS Model Specialized to GPCR The detailed biological picture is reduced to the simpler BOIS picture. This picture places the additional constraint on the BOIS model that the OFF states are pooled into a common state C . The complex C contains all the ligand-bound receptor states other than those associated with switch output. The ligand state is denoted by L , while the ligand-free receptor state is $R_{F}$ . The phosphorylation-dephosphorylation cycle (PdPC) outputs are denoted by $P_{i}$ . Switch $P_{i}$ is ON if the site on the β arr is phosphorylated, otherwise it is OFF. The GTPC is ON if the GTP-bound $G_{\alpha }$ subunit is detached from the GPCR complex, otherwise it is OFF. Nucleotide exchange provides the source J of chemical flux, which is divided equally into chemical fluxes for each switch according to the BOIS model. All ON switch states, $G_{\alpha }$ GTP and $P_{i}$ , have the same concentration also according to the BOIS model. Whether a switch is ON or OFF is determined by the reaction rate from the output to state C . This is determined by dephosphorylation in PdPCs and by dissociation in the GTPC.

We specialize the BOIS model to a GPCR complex illustrated in **Figure 3A**. Again, the time-average approach is most useful and convenient. We imagine that a single GPCR complex transitions among its allowable internal states over time. The complex is like a pulsating cloud in which all flexible states of the complex are visited by $R_{T}$ receptors. High-energy rigid states do not fluctuate as readily as flexible states.

In most cases, the point of ligand engagement is a ligand-binding pocket in the seven-transmembrane (7TM) G protein coupled receptor (GPCR) that is accessible from the extracellular fluid. Simultaneous to the ligand binding, a ternary reaction binds guanosine di-phosphate (GDP) -bound heterotrimeric G protein to the intracellular side of the complex [Lefkowitz (49)]. Ligand binding is further allosterically stabilized by the heterotrimeric G protein complex that binds to an intracellular binding pocket in the GPCR [Lefkowitz (49); De Lean et al. (48)]. This G protein complex, at this stage, has its $G_{\alpha }$ subunit bound to Guanosine Diphosphate (GDP). From the extracellular pocket, the endogenous ligand or possibly drug agonist controls signaling to downstream pathways re-conformation of the transmembrane and intracellular alpha helices of the GPCR [Wingler et al. (50)]. The conformations determine switch configurations. The switch responsible for the control of the $G_{\alpha }$ pathway is a GTPC ([39]).

The G protein is composed of three sub-units, $G_{\alpha }$ and a $G_{\beta \gamma }$ dimer. Nucleotide exchange occurs and the G-protein bound GDP is replaced with a bound guanosine tri-phosphate (GTP). This destabilizes the complex leading to the release of $G_{\alpha }$ GTP into the intracellular fluid where it activates G-protein effector coupling. This is the GTPase switch. The $G_{\beta \gamma }$ dimer is involved with other effector coupling, including β arrestin ( β arr) pathways. The C tail of the GPCR contains a number of phosphorylation sites [Latorraca et al. (5)]. These sites can be ACTIVATED and act as PdPCs, switches that leads to effector coupling of β arr pathways.

The GPCR acts as a Guanine-nucleotide Exchange Factor (GEF) that facilitates the exchange of GDP bound to the $G_{\alpha }$ subunit of the G protein complex for GTP [Coleman et al. (51); Sprang (52)]. The heterotrimeric G protein separates into two subunits, the $G_{\alpha }$ subunit bound to GTP and a βγ dimer [Liang et al. (53)] Both the $G_{\alpha }$ subunit and the released dimer transduce the signal initiated by ligand binding to the GPCR.

The other transducer, β arrestin ( β arr) is controlled by multiple PdPCs that can desensitize the $G_{\alpha }$ pathway or activate pathways downstream of β arr ([38,40]). Here, β arr can bind to the receptor core and sterically desensitize the $G_{\alpha }$ pathway. The transducer β arr can also form a megaplex by binding through phosphorylation sites to the C tail of the GCPR leading to possible continued activation of $G_{\alpha }$ pathways [Thomsen et al. (54)]. Downstream β -arrestin pathways can also be activated by the phosphorylation configuration on the C tail [Latorraca et al. (5)]. In this study, we focus on the information flow through the megaplex rather than $G_{\alpha }$ desensitization.

The C terminus of the G protein-coupled receptor binds to the βγ dimer (55]) and facilitates the phosphorylation of the C terminus and intracellular loops of the GPCR. After phosphorylation, β arrestin binds to the phosphorylated barcode and intracellular loops formed by the placement of phosphates on the intracellular portions of the GPCR. The β arrestin provides a scaffold for downstream pathways. [Smith et al. (56)] The β arrestin protein takes on many conformations with each independent conformation associated with a downstream pathway ([30]). Each conformation corresponds to a distinct configuration of PdPC switches. The GPCR is dephosphorylated by a phosphatase. Free energy is cycled into and out of the process by phosphorylation-dephosphorylation [Beard and Qian (57); Qian and Reluga (27, 29, 46)] and nucleotide exchange-hydrolysis [Coleman et al. (51); Sprang (52)].

This biological model is greatly simplified by the BOIS picture illustrated in **Figure 3B**. Here, the OFF states of the switches are pooled into a common state C , that is ACTIVATED by ligand L binding to free receptors $R_{F}$ . In this picture, the OFF state for a given switch is characterized by an absence of receptor in the ON state $R_{i}^{*}$ . The arguments stemming from MIST are unchanged and still hold in the specialized case. Moreover, the equations for MREP, Eqs. 14 through 14, remain unchanged. The key properties of the general BOIS model are preserved in this specialized configuration, including a common value for the flux in each ACTIVE switch and the constancy of the ON-state receptor concentration for each switch.

Most of the complication of the biological model is included in a single state C, which acts as a flux source J and sink for the switch outputs which are the phosphorylation sites on the C-tail and intracellular loops of the GPCR complex and the release of GTP-bound $G_{\alpha }$ . State C represents all of the ligand-bound GPCR complex other than the switch outputs: other than the concentrations of the phosphorylation site outputs and the concentration of the GTP-bound free $G_{\alpha }$ subunit. Free energy is cycled into and out of the process by phosphorylation-dephosphorylation [Beard and Qian (57); Qian and Reluga (29); 27, 46)] and nucleotide exchange-hydrolysis [Coleman et al. (51); Sprang (52)]. In the BOIS model, the ON/OFF state of the switches is determined by dephosphorylation in the PdPCs and by hydrolysis of GTP in the GTPC.

The GTPC switch may be more complicated than the PdPCs. Here, association and dissociation of $G_{\alpha }$ adds some complication as does the presence of hydrolysis and its catalyst RGS. The value of the free $G_{\alpha }$ concentration also has an effect. This added complication does not affect the results and conclusions here.

The number of receptors bound to ligands depends on the free-ligand concentration. If the receptors have not been exposed to ligands and the current free-ligand concentration is zero, then, obviously, the number of ligand-bound receptors is zero. As the free-ligand concentration increases, ligands become bound to receptors. This is a dynamic process with ligands associating and dissociating with the receptors. If the addition of free ligands is slow enough, the ligand-bound receptor concentration achieves a steady state that depends only on the ligand concentration. In other words, the bound receptor concentration changes adiabatically with ligand concentration. When the ligand concentration becomes very large, all receptors become bound.

No information is transmitted by the ligands when the number of ligand-bound receptors is zero or when all receptors are bound. In these two cases, the information H stored in the bound-receptor probability distribution is zero. The maximum storage of one bit occurs when half the receptors are ligand-bound. Any information transmission rate greater than one bit must be supplied by another mechanism than the simple ligand binding described here.

It should be noted that reversing the ligand population from a high value to zero does not necessarily reproduce the same ligand dependence on the number of bound receptors as in the forward case. This is due to the fact that once a receptor is bound to a ligand, it can change into other states such as, for instance, a ligand-bound phosphorylated state, which may not have the same ligand dissociation constant as the unphosphorylated state. One then expects hysteresis in the forward and reverse trajectories of the bound receptor concentration. We are not aware of any experiments that have examined the removal of free-ligand concentration from a population of fully bound receptors. We do not believe that the hysteresis prediction has been tested.

The kinetics of the model is described by the following ordinary differential equations (ODE) where we designate the state concentrations by the name of the state


(24) $$\frac{dR_{F}}{dt}=k_{L}^{+}C-k_{L}^{-}R_{F}L$$

(25) $$R_{T}=R_{F}+C+\sum_{i=1}^{N}R_{i}^{*}+R_{G}^{*}$$

(26) $$\frac{dR_{i}^{*}}{dt}\;=\;J_{0}\;-\;k_{i}^{-}R_{i}^{*}$$

and


(27) $$\frac{dR_{G}^{*}}{dt}=J_{0}-k_{G}^{\;}R_{G}^{*}$$

where N is the number of ACTIVE phosphorylation switches and the k notation indicates reaction rates. All receptor concentrations are normalized to $R_{ref}$ from Eq. 10. Here, $R_{i}^{*}$ is the ON-state concentration of the of the PdPC and is equal to the probability that the PdPC is ON, while $R_{G}^{*}$ is the same quantity for the GTPC.

Simulations were performed in which the ligand concentration was initially set to zero. The concentration was increased until all receptors were bound. At that point, all ON switch concentrations were at their common maximum value.

For a finite value of the chemical flux $J_{0}$ , some initial conditions lead to non-physical results. For instance, in the relevant case in which all receptors are initially in the free state $R_{F}$ and none are in any ligand-bound state, Eqs. 26 and 27 immediately drive the ON-state concentrations $P_{i}$ and $R_{G}$ to negative values, which is non-physical and not permitted. This implies that in the state in which the complex encounters the ligand for the first time, all switches are INACTIVE and contain no chemical flux ( $J_{0}=0$ ). Simulations indicate that no physical solutions are possible until the ligand concentration increases to the level at which the complex concentration C is equal to the number of receptors that will turn on one switch ( C=1 ).


(28) $$C=\frac{LR_{T}}{L+K_{L}}=1$$

(29) $$K_{L}=\frac{k_{L}^{-}}{k_{L}^{+}}$$

**Figure 4:**
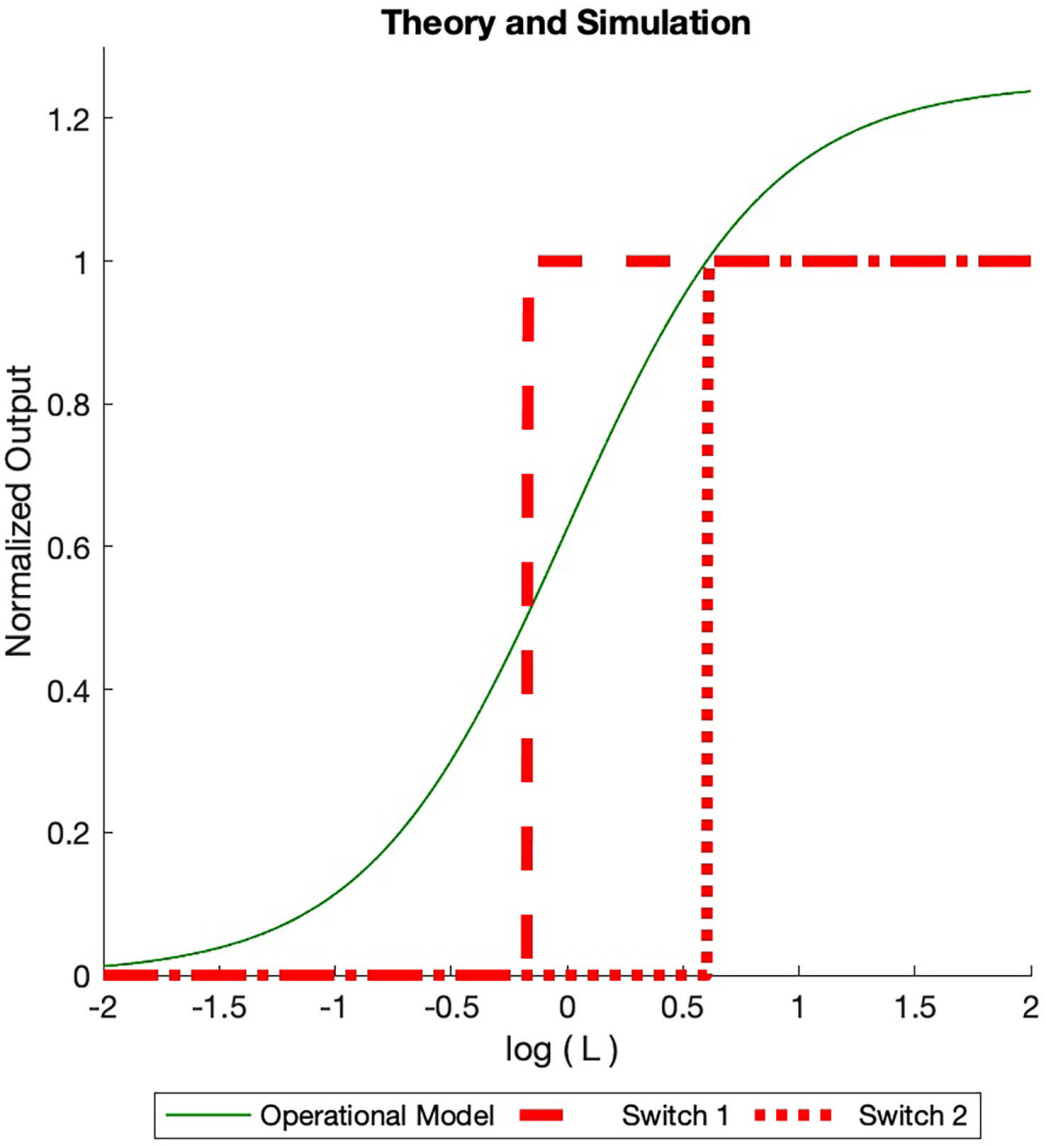
Dose Response Simulated (BOIS) (red) and Operational (solid green) models dose response curves are displayed. The total receptor concentration is $R_{T}=2.5$ in normalized units. A maximum of $2<R_{T}$ ON switches are allowed. For each ON switch, there is an OFF switch. The ligand concentration increases from the left and is normalized to the ligand-receptor dissociation constant. For small initial ligand concentrations no switches are ACTIVATED in the BOIS model. When the ligand concentration in state C increases to one, two switches become ACTIVATED (Eq. 28), ON and an OFF switches. Either a GTPC switch is activated and turned ON (solid black line) or a PdPC is ACTIVATED and turned ON (dotted black line). When the switches are ACTIVATED, the concentration in state C drains into the switch states. As the concentration is state C increases to one once more, four switches become ACTIVATED and two turn ON. The switch always achieves the same receptor concentration when ON, however the ligand concentration at which the switch turns ON varies. For higher receptor concentration $R_{T}$ , more ON switches are possible with a maximum number equal to the largest integer less than the normalized receptor concentration. In contrast, the Operational model varies with the number of switches. We plot the curve for two switches assuming the receptor concentration is equipartitioned between the two. Once the BOIS model is ACTIVATED and the switches are turned ON, then the system does not return to the INACTIVATED state. If the ligand concentration goes to zero then the ON state turns OFF for the Operational model, in contrast to the BOIS model.

At that point one switch can be ACTIVATED and turn ON. The concentration goes to zero at this point. As the ligand concentration increases more, the receptor concentration C once again increases to a value of one at which a second pair of ON/OFF switches are ACTIVATED. This process continues until the total number of INACTIVE switches is ACTIVATED or the ACTIVE switches have consumed the total number of receptors. This is illustrated in the simulation runs in **Figure 4**.

This argument highlights the important transition from inactivated switches to activated switches as free-ligand concentration increases in the presence of receptors that are not initially bound to ligands. At low free-ligand concentrations, the total flux into the system is zero. No switches are ACTIVE. The complex is not capable of sending information to downstream processes. As free-ligand concentration increases, however, a point is reached when a phase transition occurs and the chemical flux becomes finite. At this point, the complex is able to transmit information. This supports prediction (3) in the abstract.

The sharp transition between ON and OFF states illustrated in **Figure 4** is an artifact of the theory. In physical phase transitions and in all theories based on the mean-field-theory approximation, as is the BOIS model, fluctuations spread sharp transitions. When fluctuations are included in the theory, the transitions become smooth [Uzunov (58)].

An assumption of the BOIS model in Eqs. 24 - 27 is that the only state in which a receptor can dissociate from a ligand is C . This is consistent with many models, including the Operational Model. The dissociation constant from phosphorylated states, for instance, is zero. An important implication is that, in the BOIS model, once a switch has been activated it does not become deactivated if the free-ligand concentration decreases to zero. The ligands bound to the receptors in the switches remain bound. A process from outside the model must deactivate the switches. It must be terminated by an external process such as arrestin blockage. This is consistent with [Tran et al. (59); Gurevich and Gurevich (60); Wilden et al. (61); Lohse et al. (62)].

Both the Operational model and the BOIS model treat much of the receptor complex as a black box represented by state C in Eq. 24. The Operational model differs from the BOIS model in that the constant flux $J_{0}$ in Eqs. 26 and 27 is replaced by fluxes $k_{i}^{+}C$ and $k_{G}^{+}C$ , respectively. The specialized BOIS model makes a very different set of predictions from the Operational model. There is no concept of ACTIVE and INACTIVE switches in the Operational model as there is in TCM. The flux in a switch can take on any values given by $k_{i}^{+}C$ and $k_{G}^{+}C$ . In the scenario in which all ligands are free and the ligand concentration is increased from zero, a single switch becomes saturated when it has absorbed the entire number of receptors $R_{T}$ . If multiple switches are present in the system, the response is partitioned equally among the switches if they are identical. This is illustrated by the green curve in **Figure 4**. If the number of switches changes, then so does the maximum response.

Importantly, in the Operational model, the saturated outputs drop to zero if the free-ligand concentration drops back to zero. We can see this quantitatively by noting that the Operational model for the system describes the NESS by [Black and Leff (7)].


(30) $$R^{*}=\frac{K_{S}L}{L+K_{L}+K_{S}L}R_{T}$$

Here, $R^{*}$ is the ON concentration of the switch and $K_{S}$ are given by


(31) $$K_{S}=\frac{k^{+}}{k^{-}}$$

for a switch. Note that the Operational model, Eq. 30, depends continuously on ligand concentration and linearly on the total receptor concentration $R_{T}$ . This is quite different from the BOIS model, which is step-wise dependent on the ligand concentration. The Operational model increases the ON concentration for all switches simultaneously, while the BOIS model turns on one switch at a time. From Eq. 30 we see that the output concentration $R^{*}$ goes to zero as the free-ligand concentration L goes to zero.

### 2.5 Switch manipulation

Molecular switches exist within a milieu of energy fluctuations, which cause the molecular conformations to rapidly change. On the other hand, for information transmission to be useful, the conformations associated with information storage and transmission must be long-lived. [Schrodinger et al. (44)], otherwise, the information rapidly disappears. Therefore, bonds associated with the switches, whether covalent or conformational, must have lifetimes comparable to the time needed for information storage and transfer. The bonds associated with switch configurations must be rigid. In the BOIS model these rigid bonds are represented by the reaction rates $k_{i}^{-}$ and $k_{G}^{-}$ in Eqs. 26 and 27.

**Figure 5:**
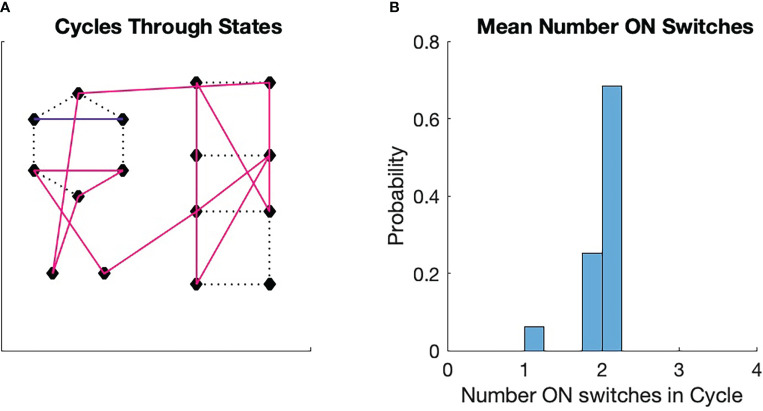
Monte Carlo simulations of switch cycles. An initial switch was chosen randomly from among all 16 switches. The transition to the next state was determined by a transition probability, which was initially uniform. if the transition between two states was chosen, the transition probability from the first to the second state was increased by 10% and then renormalized. The transition to the next state is determined in the same manner, with the consequent renormalization. The process is repeated for 16 transitions. Then a new initial state is chosen and a new trajectory is generated with modification of the transition probabilities. The process continues until a fixed point in the cycle formation is achieved. The fixed points can vary from run to run. **(A)** A fixed point for the process. We see here three stable cycles, one that contains 13 states (magenta), one that contains two states (blue), and one that is a single state. The singleton cycle has one ON switch (see **Figure 2C**). The two-state cycle has two ON switches (**Figure 2A**). **(B)** Probability that a switch chosen randomly will land in a cycle that has a given average number of ON states in the cycle. The probability for the three cycles are displayed. Note that the probabilities are clustered around the ON state number 2, which is half the number of total switches in the configuration.

MIST requires that the number of ON states is equal to the number of OFF states, at least statistically. Some indications of this have been observed experimentally [Latorraca et al. (5)]. This is a global requirement involving the entire portion of the GPCR megaplex associated with switches. The mechanism to implement this requirement is unclear but we can gain some insight with a simple simulation illustrated in **Figure 5**.

As was pointed out in the discussion of **Figure 1**, biological processes, in order to repeat actions, must exist as cycles [Kauffman (63)]. We can perform a very simple numerical experiment to see how these cycles might form from a very simple set of physical rules. We imagine a collection of four switches with states as illustrated in **Figure 2**. We then imagine that each state s transitions to another state at times t=1,2,3,⋯ with a certain transition probability p[s(t)→s(t+1)] . We then run a Monte Carlo simulation of state transitions with a single requirement: if the simulation transitions from s(t) to s(t+1) , then the transition probability for those two states is slightly increased. This type of modification to the transition probability is known as Hebbian learning and is important in the study of neural systems [Munakata and Pfaffly (64); Keysers and Perrett (65); Gerstner and Kistler (66)].

Each simulation starts with a random initial state. The requirement states that a transition from one state to another affects the conformation of the flexible protein matrix such the conformation has a physical memory, a lasting impression, of the transition. We see in **Figure 5A** that the simulation spontaneously forms cycles. Precisely which cycles are formed varies from simulation to simulation and is determined by the random number seed. We see from **Figure 5B** that the probability of a state being in a cycle in which the average number of ON states among the cycle switches clusters around two, which is half the total number of switches M=4 . We found this to be true for all the approximately 100 simulations we ran.

These results are easy to understand. If we pick a state with uniform probability from among the 16 possible choices, then the probability that the state will be associated with a particular cycle is greater the more states are in the cycle. Larger cycles, on the other hand, sample more of the total number of states than smaller cycles. As the cycles become larger, the average number of ON switches in the cycle approaches 2, which is the average number for all 16 states. This leads to the tight clustering around the average of 2 seen in **Figure 5B**.

It is well known that balance of this nature, excitatory-inhibitory balance, occurs at the cellular level [Froemke (67); Ganguly et al. (68)]. At least in some periods of neural development, the number of inhibitory and excitatory neurons are statistically equal in neuron switches. The balance in the cellular case is determined by Cl ${\;}^{-}$ concentration in the extra- and intra- cellular fluid [Ganguly et al. (68)], which, as we propose here, is a system-wide control of ON/OFF balance.

One important implication of this ON/OFF balance is that if a switch is flipped, then, at least statistically, another switch is flipped oppositely. The hypotheses generated by the arguments of this section are theoretical predictions that merit experimental tests.

## 3 Experimental tests of predictions

The BOIS model makes several predictions, some of which can be compared with experiments. In particular, the prediction that all ON-state switch concentrations are the same value for a given receptor even if different ligands are applied or the number of ON switches varies can be tested, as described by Eq. 11. The output response to ligand-concentration, **Figure 4**, can be compared with assay bias curves. This provides a mechanism for understanding and quantifying the observed values of $EC_{50}$ for the dose-response curves in bias assays ([41]). Predicted information transmission for a given number of ACTIVE phosphorylation sites can be compared with experimental information transmission experiments [Keshelava et al. (3)].

### 3.1 Bias assay experiments

**Figure 6:**
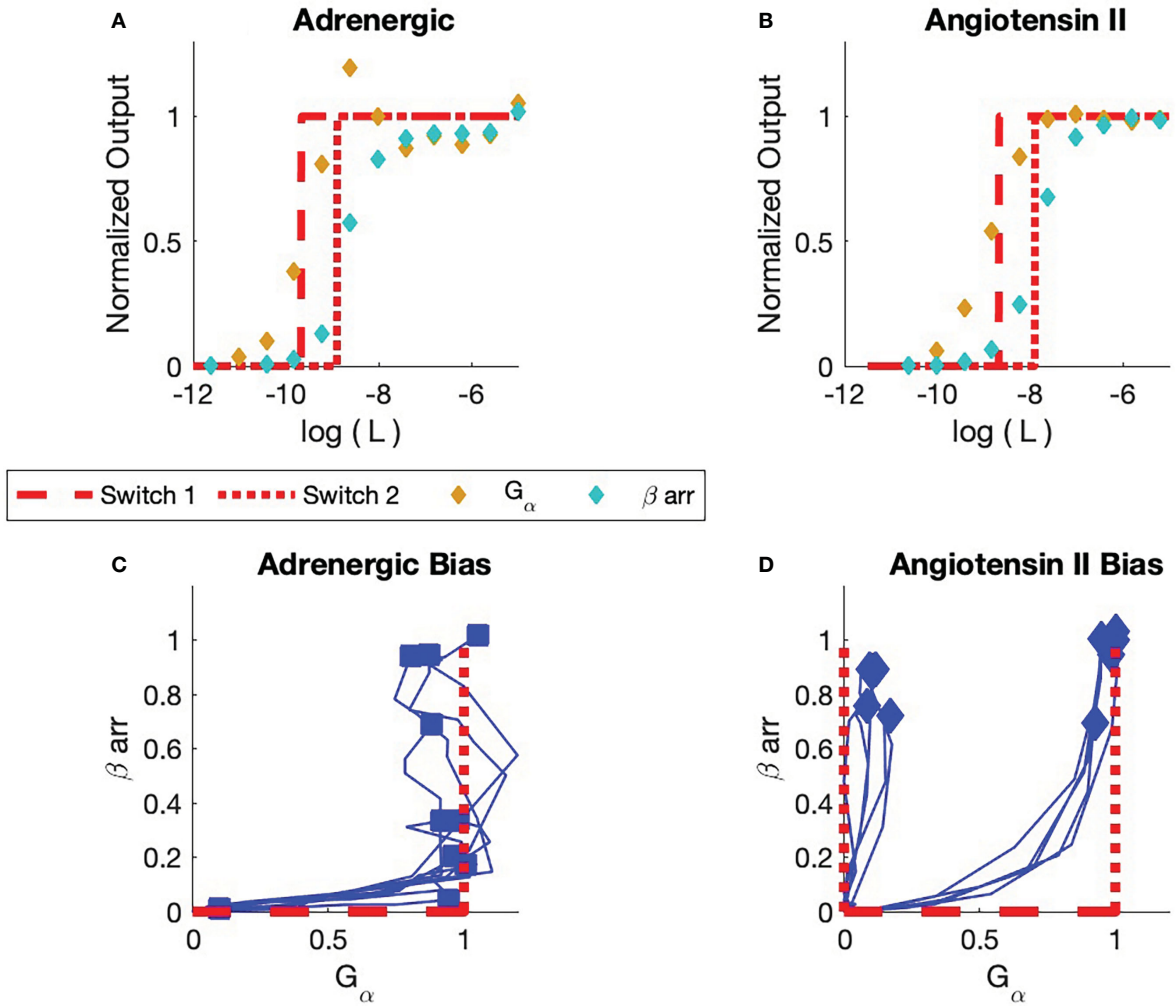
Comparison of experiment and simulation **(A)** Adrenergic Receptor with Formoterol as Ligand. The simulation is displayed in red. Receptor concentrations are normalized to the reference concentration, which for this adrenergic receptor is the maximum concentration of Formoterol. All receptor concentrations for all adrenergic assays are normalized to this reference concentration. The dashed line is the simulation of the activation of the first switch. The dotted curve is the activation of the second switch. The yellow markers are the observed assay dose response for the $G_{\alpha }$ pathway. The cyan markers are the observed assay dose response for β arr. **(B)** Angiotensin II Receptor with Angiotensin II as Ligand. The reference concentration is taken to be the maximum concentration of angiotensin II. **(C)** Bias Plot for All Ligands for Adrenergic Receptor (see **Table 1**). The simulation results are displayed in red. Note that some ligands are $G_{\alpha }$ biased; their endpoints lie close to the $G_{\alpha }$ axis. Other ligands are balanced; their endpoints lie at (1,1). No β arr bias is seen in this set of ligands. **(D)** Bias Plot for All Ligands for angiotensin II Receptor. This plot illustrates balanced bias and β arr bias. For balanced bias the first switch is the GTPC and the second switch is the PdPC that activates arrest in recruitment. For β arr bias, the GTPC is not activated. The BOIS model predicts that the first ACTIVATED is a PdPC that is not observed. Here, $G_{\alpha }$ bias is only seen for ligand concentrations that are associated with sub-maximal response.

We compare the assay data from [Rajagopal et al. (1)] with simulations based on the BOIS model in **Figure 6**. Assay results on two different receptors, β2 adrenergic ( $\beta_{2}AR$ ) and angiotensin II type 1A ( $AT_{1A}R$ ), for which IP and cAMP, respectively, were measured as an indirect proxies for these switch ON states, are displayed. The G proteins were in the $G_{q}$ class for $\beta_{2}AR$ and in the $G_{s}$ class for $AT_{1A}R$ . The study also measured recruitment of β arrestin 2.

Two different downstream pathway concentrations were measured for each receptor, the $G_{\alpha }$ pathway and one β -arr pathway. Other pathways may have been present, but they were not measured. The estimated reference receptor concentration $R_{exp}$ was taken to be the maximum Formoterol concentration for the adrenergic receptor and angiotensin II maximum concentration for the angiotensin II receptor. The maximum downstream concentrations normalized to $R_{exp}$ for each pathway are given in columns marked $G_{\alpha }$ and β arr in **Table 1**. The $EC_{50}$ concentrations in the table are measured in moles per liter. Several distinct ligands, listed in **Table 1**, were applied to each receptor.

**Table 1:** Assay data.

Adrenergic					Angiotensin II				
	*G_α_*	log(*EC_50_*)	*β* Arr	log(*EC_50_*)		G_α_	log(*EC_50_*)	*β* Arr	log(*EC_50_*)
Form	1.05	-9.62	1.02	-8.61	TRV120056	0.95	-7.34	1.00	-6.42
Iso	0.87	-9.71	0.94	-8.14	TRV120055	1.00	-7.97	1.03	-7.05
Fen	0.80	-9.41	0.94	-7.81	AngII	1.00	-8.84	1.00	-7.90
Epi	0.88	-9.01	0.69	-7.25	S1C4	0.93	-8.84	0.70	-6.66
Salb	0.92	-8.30	0.33	X	A1	0.98	-8.52	0.95	-7.68
Salm	0.97	-8.26	0.33	X	TRV120034	0.12	X	0.89	-7.68
Clen	1.00	-8.85	0.17	X	TRV120026	0.09	X	0.89	-6.64
Norepi	0.96	-6.84	0.21	X	TRV120045	0.11	X	0.89	-7.57
Dob	0.94	-6.57	0.04	X	SGG	0.17	X	0.72	-5.69
Pind	0.10	X	0.01	X	TRV120044	0.09	X	0.76	-6.79

Assay values for maximum stimulus for adrenergic and angiotensin receptors [Rajagopal et al. (1)]. The concentrations Gα and β arr are normalized to the reference value Rref. Ligand concentrations are molar. The ligand names correspond to the names used in [Rajagopal et al. (1)]. Balanced ligands for the adrenergic receptor are Formoterol, Isoproterenol, Epinephrine, and Fenoterol. Balance ligands for the angiotensin II receptor are Angiotensin II, TRV0120055, TRV0120056, A1, and S1C4. Adrenergic ligands that are Gα biased are Dobutamine, Norepinephrine, Clenbuterol, Salmeterol, and Salbutamol. No adrenergic ligands in the sample are β-arr biased. Angiotensin II ligands that are β-arr biased are TRV0120044, TRV0120045, TRV0120034, and S1G4G8. No angiotensin ligands in the sample are Gα- biased.

Experimental dose-response and bias curves from [Rajagopal et al. (1)] are displayed in **Figure 6**. All concentrations are normalized to the maximum observed concentrations $R_{exp}$ for each receptor. Here, $R_{exp}$ is the maximum concentration of Formoterol for the adrenergic receptor; it is the maximum concentration of angiotensin II for the angiotensin II receptor. Figure A displays the $G_{\alpha }$ and β arr recruitment responses to Formoterol and angiotensin II for the adrenergic and angiotensin II receptors, respectively.

**Figure 6A** illustrates the two dose response curves for the ligand Formoterol binding to the adrenergic receptor. The $G_{\alpha }$ response is indicated by gold markers and the β arr response is indicated by cyan markers. The maximum normalized Formoterol response concentration is one since $R_{exp}$ is equal to the maximum Formoterol concentration.

The BOIS model is indicated in red. We assume that four switches are involved in the process. As the ligand concentration L increases from zero in the presence of a receptor that has not been exposed to the ligand before, the theoretical response is zero. The chemical flux through all the switches is zero. When the normalized bound-ligand concentration in the receptor (state C) reaches one, the first switch turns on (dashed curve). We set the $EC_{50}$ for the first switch of the BOIS model to be the $EC_{50}$ of the first switch measured in the experiment. The receptor becomes activated. A chemical flux flows through the receptor states. The ligand concentration in receptor states associated with the body of the GPCR, state C, approaches zero and all the receptor states are associated with the first switch that turns ON. As the ligand concentration increases further, the concentration in the body of the GPCR complex increases once more until the second switch (dotted) turns ON. The $EC_{50}$ of this switch in the BOIS model is determined by the $EC_{50}$ of the first switch.

For Formoterol applied to the adrenergic receptor, the experimental observations indicate that the first switch that turns ON is the $G_{\alpha }$ (gold) response. The second switch is the observed β arr recruitment (cyan) switch. This can be seen also to be true for all ligands tested with the adrenergic receptor in **Figure 6C**. Here, we have reproduced the bias curves (blue) from Rajagopal et al. (1). Each curve represents a unique ligand given in **Table 1**. The markers indicate the maximum output for each ligand and receptor. The horizontal axis measures the output to the $G_{\alpha }$ or GTPC pathways, while the vertical axis measures the output to the β arr PdPC pathway. We see that all responses start as zero as would be expected. All measured ligands turn the $G_{\alpha }$ switch ON first and then some of the ligands also turn on the β arr recruitment switch. It is important to note that the maximum responses all lie roughly around the points (0,0), (0,1), and (1,1). This is support for the BOIS prediction that all responses saturate at a common concentration. The biological interpretation is that the ligand-bound receptor first binds to the G protein. Then, the receptor can stay in that state with the β arr downstream pathway untriggered or the ligand/G protein-bound receptor can facilitate the recruitment of β arr.

**Figure 6B** displays the response curve for Angiotensin II ligand on the angiotensin II receptor. As was the case with the adrenergic receptor, the first switch to turn ON for the angiotensin II receptor is a $G_{\alpha }$ switch as seen in **Figure 6B**. The second switch is associated with β arr. The interpretation of the bias curves **Figure 6D** is not as simple as the interpretation of the bias curves for the adrenergic receptor, **Figure 6C**. We note that if a receptor triggers the $G_{\alpha }$ response it also triggers a β arr response at a higher ligand concentration. We see that the right blue curve in the figure first passes near the (0,1) state before proceeding to the balanced (1,1) state. This indicates, within the context of the BOIS model, the $G_{\alpha }$ switch is turned on first and then all receptors that have the $G_{\alpha }$ switch turned ON transition into the β arr recruitment state. In more biological language, this means that the ligand-bound receptor first binds with G protein, which facilitates the recruitment of β arr. The left blue curve in **Figure 6D** indicates that the β arr can occur without the intermediary of the measured $G_{\alpha }$ pathway. In other words, the measured $G_{\alpha }$ pathway does not facilitate β arr recruitment. The β arr recruitment occurs with an

$EC_{50}$ of the second switch. Within the context of the BOIS model, this indicates that an unmeasured switch was the first to turn ON and that this unmeasured switch may have facilitated the recruitment of β arr.

We see that the saturated responses, the ON states, for the experimental assays are clustered about the points (0,1), (1,1), and (1,0) in the bias curves. One ineffective measured ligand had a response of (0,0). This point is not displayed. This indicates that the common saturated-concentration value of *R_exp_* is the common concentration for all the ligands, for multiple numbers of ON switches. The BOIS predictions indicate the this number is $R_{ref}$ . We can conclude that the measured value $R_{exp}$ and the BOIS value $R_{ref}$ are equal.

The fits to data in **Figure 6** rely on just three free parameters, $R_{ref}$ , the $EC_{50}$ for one switch, and ΔR (see Eq. 7). Each of the three parameters has an intuitive meaning.

The Operational model and TCM predict different maximum response for different numbers of ON switches. This is not observed in the observations in **Figure 6**. The Operational model and TCM predict that all switches turn on simultaneously. This also, is not observed. This defect of the Operational model can be corrected in a more elaborate Mass-Action model that introduces additional degrees of freedom for reaction rates that vary the $EC_{50}$ for each switch. The BOIS model, however, makes the prediction with a paucity of free parameters.

### 3.2 Information-flow experiments

Recent single-cell experiments on the muscarinic acetylcholine receptor M3R [Keshelava et al. (3)] indicate that more than two bits of information is transmitted through the receptor complex. In other words, the channel capacity $C_{I}$ was measured to be greater than two bits. This is significantly higher than previous population-level experiments that measured transmission at about one bit (see Keshelava et al. (3) and Zielińska and Katanaev (2) for references) The difference is attributed to extrinsic noise, that is inter-cellular noise, in the cell population. Increased information flow is observed when measurements are made at the cellular level as opposed to the population level. Accurate measurement of information flow seems to be sensitive to the resolution of the experiments.

The authors point out that a capacity slightly greater than two allows for four possible downstream effector couplings, which allows for ligand bias. Bias is measured in Rajagopal et al. (1). The BOIS model predicts that for four switches, the capacity is $C_{I}=\log\;(6)=2.58$ bits, which is consistent with both the single-cell capacity measurements and the bias experiments.

## 4 Discussion

The approach of this study is mathematics/physics-based, which is not common in biology in the manner it is used here. The approach posits an idealized thought experiment. The thought experiment itself does not contain all the richness of reality, but it attempts to create an imaginary world that can be used for prediction. The goal of a thought experiment is to generate hypotheses that can be tested by existing or future experiments. The implications of the thought experiment are explored and predictions are compared with experimental observations. Most scientists would agree that biological systems are constrained by the laws of physics and mathematics, so it is not only useful, but imperative, that these constraints be examined in biological contexts.

The thought experiment here takes as its fundamental assumption that biological systems are optimized. Biologists often point out that components of biological systems such as enzymes are not optimal. By this, they may mean that the system under study does not seem to operate most efficiently in regards to energy consumption. This does not mean that the system is not optimized according to some different criteria consistent with natural selection. In this study, we postulate that biological systems have optimized information and entropy flow and we then determine the implications. We chose these quantities to optimize because they seem reasonable from the perspective of natural selection. There may be other reasonable choices.

This study examined an ensemble of energy consuming switches. We imagined that natural selection chose a system that transmitted information perfectly and that evolved toward thermal equilibrium along the shortest path subject to mass and free-energy flow through the ensemble. Maximization into a stable system required a balance between protein flexibility and rigidity [Schrodinger et al. (44)].

Some of the assumptions and predictions of the BOIS model have experimental support. The picture of an array of switches is supported by measurements of the phosphorylation sites on the isolated C terminus of the GPCR [Latorraca et al. (5)]. Measurements of information flow in individual cells, as opposed to tissue measurements, indicated that the information transmission through the GPCR is greater than 2 bits [Keshelava et al. (3)]. This is consistent with the BOIS model that predicts that the information transmission is log_2_ (6)=2.58 bits for four switches. The common receptor reference concentration $R_{ref}$ was observed in (1, for example). The steady-state and transient bias curves predicted by the BOIS model were observed in bias curves [Rajagopal et al. (1)]. The BOIS model requires the arrestin-bound C tail of the GPCR complex to be flexible such that distinct phosphorylation sites can come in contact with each other and also the GTPC. This is supported by observations [Kahsai et al. (69); Latorraca et al. (4); Eichel et al. (70)].

The BOIS model describes the transition between an inactive GPCR complex that is incapable of transmitting information to one that can transmit information as free-ligand concentration increases in the presence of receptors that are not initially bound to ligands. The model predicts a phase transition in which the chemical flux in the system turns on suddenly as free-ligand concentration increases.

The BOIS model indicates that the number of OFF switches is statistically equal to the number of ON switches if the system is operating at maximal efficiency for information flow. The tendency of the switch configurations to have a constant number of ON switches was observed in isolated C tails of the GPCR complex [Latorraca et al. (5)]. We speculated on one possible mechanism for this behavior in Sec. 2.5 where we showed that self-reinforcing transitions through the various switch configurations lead to statistical equivalence of the number of ON and OFF switches. The trajectories through the state space formed cycles as one would expect for biological processes that are able to repeat their actions. Moreover, perturbation of this system with the addition of a ligand actually disrupts the equality of the number of OFF/ON switches, at least briefly. A perturbation sets in motion a set of changes that bring the system back to OFF/ON equality. Similar behavior at the cellular level is seen with neurons in excitatory/inhibitory balance in various stages of development [Froemke (67); Capogna et al. (71)]. As such, optimal information flow requiring this OFF/ON equality may increase the fidelity of signaling. In fact, switch chains of this sort have been suggested as a means to enhance temporal cooperativity among the switches [Qian (46)].

We are not aware of experiments that can be compared with other BOIS predictions. For example, we do not know of currently performed experiments that can test the BOIS assertion that the chemical flux in each switch is constant, nor have we seen experiments that identify the return reaction rates, $k_{i}^{-}$ and $k_{G}^{-}$ in Eqs. 26 and 27, as the target to change switch states between ON and OFF. We have not seen GPCR experiments that address the prediction that the number of ON switches is statistically equal to the number of OFF switches (Sec. 2.5). Finally, we are not aware of experiments that determine whether the ON states turn OFF if the extracellular ligand concentration drops to zero (Sec. 2.4). These unverified predictions comprise a set of hypotheses for testing.

The most potentially practical applications of the BOIS model may be for drug discovery [see Zhuang et al. (72)] for a very recent relevant study of bias]. The BOIS picture is a systems picture for the selection of downstream pathways. The BOIS model is not able to supply a recipe for drug discovery. It does, however, show some promise to point out meaningful search pathways. Kinases, for example, are currently receiving more research attention than phosphatases. This study suggests that, for bias control, it may be necessary to increase attention paid to phosphatases.

Current drugs operate on coarse scales. For instance, Angiotensin Receptor Blockers (ARB), among other things, affect blood pressure and renal glomerular damage by blocking all the information transfer in the Angiotensin II ( $AT_{1}$ ) receptor. If, hypothetically, blood-pressure control lies on one $AT_{1}$ pathway and renal-function control lies along another pathway (Ruiz-Ortega et al. (73), then ARBs block both pathways indiscriminately. Precision medicine will require knowledge of bias control at a much finer scale than is currently practiced. In that case detailed knowledge of the order and how switches are turned on with varying free-ligand concentration is crucial to controlling which switches are ON and OFF. The discussion around **Figure 6** illustrates how the BOIS model might inform the fine temporal application of drugs that affect multiple pathways. In the case of angiotensin II receptor $AT_{1A}$ , for instance, the BOIS model suggests that at least two distinct pathways lead to β arr recruitment, one in which the $G_{\alpha }$ pathway is crucial in the recruitment and another pathway in which it seems that recruitment depends on a switch other than the $G_{\alpha }$ switch. The identification of pathways that be controlled by different mechanisms may, for instance, be important for the treatment of diabetic kidney disease in which the angiotensin receptor seems to have downstream pathways that affect cardiovascular response and other pathways that affect the kidney more directly.

In another example, it should become possible to deliver current opioids automatically into the tissue as micro-doses in a continuous or dynamic prescribed regime such that the anti-nociceptive properties of that specific drug are realized whereas the addictive side effect is avoided.

The BOIS model suggests that the number of ON and OFF switches are statistically equal (Sec. 2.5). This implies that flipping a switch may affect the switch state of another switch or switches that may be physically distant from the flipped switch. This is a system-wide effect that seems to emerge from the BOIS picture of rigid bonds embedded in a matrix of flexible protein. In that case, the concept of drug targets must be expanded to include the manipulation of the entire protein matrix rather than just manipulation of specific bonds.

The BOIS model may have implications for systems biology. The BOIS model relies on protein flexibility for reaction binding energies less than approximately 12 kcal/mol , the amount of energy per reaction from ATP [(45, Sec. 15.2) Qian and Reluga (29)], and rigid protein reactions for binding energies greater than this number. This model differs significantly from models such as mass-action models in which proteins are completely rigid and inflexible, and reaction rates are constant. The main variables in the BOIS model are chemical fluxes in each reaction, while the key variables in models such as mass action are concentrations of each chemical species. Because of protein flexibility, the BOIS model is more like a fluid than a rigid reaction network. The BOIS model behaves globally to optimize external forces just like water forms a flat surface in response to gravity.

The BOIS model suggests a physics laboratory in which to test conjectures in nonequilibrium thermodynamics. The principle of MREP is much less well-established than the Second Law of Thermodynamics [Jaynes (12)]. The bias and information-transmission experiments [Rajagopal et al. (1); Keshelava et al. (3)] can be thought of as physics experiments in nonequilibrium thermodynamics.

## Data availability statement

The data analyzed in this study is subject to the following licenses/restrictions: All data used in this study has been previously published by other authors. Requests to access these datasets should be directed to Dr. Sudarshan Rajagopal, sudarshan.rajagopal@duke.edu and Ron Dror, ron.dror@stanford.edu.

## Author contributions

RJ performed the analysis and wrote the manuscript. AJ edited the manuscript. All authors contributed to the article and approved the submitted version.
