# Supplementary material for: Model of ligand-triggered information transmission in G-protein coupled receptor complexes
Source: Front Endocrinol (Lausanne). 2023 May 9;14:1111594. doi: 10.3389/fendo.2023.1111594 (PMC10286511; doi:10.3389/fendo.2023.1111594)
Supplement: Supplementary file 1 [file DataSheet_1.pdf]

Supplementary Material

0.1 Information Transmission in a Single Switch

Information transmission in biological switches is well studied Qian and Roy (2012); Selimkhanov et al. (2014). The information capacity C_i of a switch i, the maximum amount of information that can pass through a switch, has been calculated for the reaction

$$[\mathrm{R}] \underset{\alpha^{-}\ [\mathrm{K}]}{\overset{\alpha^{+}\ [\mathrm{K}]}{\rightleftharpoons}} [\mathrm{R}^{*}] \tag{S1}$$

$$[\mathrm{R}] \underset{\beta^{-}\ [\mathrm{P}]}{\overset{\beta^{+}\ [\mathrm{P}]}{\rightleftharpoons}} [\mathrm{R}^{*}] \tag{S2}$$

Here, $[K]$ and $[P]$ are kinase and phosphatase concentrations respectively. The forward and back reaction rates are $k^+ = \alpha^+[K]$ and $k^- = \beta^+[P]$, respectively. In equilibrium, the forward and microscopically reversed rates are in detailed balance as are the back and microscopically reversed rates. This is not true in biologically relevant situations. In that case the forward and back rates are much larger than the respective microscopically reversed rates. The further into nonequilibrium the process is, the more efficient is the information transmission. To see this, note that the capacity , the maximum information flow, in a switch is given approximately by Qian and Roy (2012)

$$C_i \approx \frac{\alpha^+}{\alpha^+ + \alpha^-} - \frac{\beta^-}{\beta^+ + \beta^-} \tag{S3}$$

The capacity C_i in the switch is maximized when the reaction rates α^+ and β^- in the clockwise direction is much greater than the microscopically reversed rates α^- and β^+ in the counter-clockwise direction. This is just the condition for the process to be far from equilibrium. In equilibrium, the forward rates are equal to the reverse in detailed balance.

$$\alpha^+ = \alpha^- \tag{S4}$$

$$\beta^- = \beta^+ \tag{S5}$$

The further the rates are from detailed balance, the further the process is from equilibrium. The process is driven far from equilibrium by free energy forced into the system that is in excess of the thermal energy from the heat bath.

The free-energy surplus is a result of the concentration of ATP being held out of equilibrium with its reactants ADP and phosphate Qian and Reluga (2005). Under conditions in the cell, this energy is about $12\ kcal/mol$ (Berg Jeremy et al., 2019, Sec. 15.2) much higher than the thermal energy of $0.6\ kcal/mol \approx 1/40\ eV$ and much lower than the energy required to break covalent bonds, about $100\ kcal/mol \approx 4\ eV$. For these energies, the reaction rates obey the condition Qian (2007)

$$\frac{\alpha^+\ \beta^-}{\alpha^-\ \beta^+} \approx 10^{10} \tag{S6}$$

which, from Eq. S3, indicates that the capacity is quite close to its maximum loss-free value for biological processes. For biological systems driven by ATP, switches are nearly loss-free.

0.2 Maximization of Entropy Production

0.2.1 General BOIS Model

We assume one ACTIVE GTPC and N ACTIVE PdPCs such that $M = N + 1$. The steady-state entropy production rate σ for all the switches is Qian (2003)

$$T\,\sigma = -\sum_{i=1}^{N} J_i\,\mu_i - J_G\,\mu_G \tag{S7}$$

$$J_i \;=\; \Pr(\,ON \mid i\,)\,k_i^- \;=\; \Pr(\,OFF \mid i\,)\,k_i^+ \tag{S8}$$

$$J_G \;=\; \Pr(\,ON \mid G\,)^2\,q_G^- \;=\; \Pr(\,OFF \mid G\,)\,q_G^+ \tag{S9}$$

where T is the temperature of the heat bath in energy units, J_i is the chemical flux of a single receptor passing through the receptor states of PdPC i, and $\mu_i \le 0$ is the free-energy cost for a receptor to pass through the PdPC, with similar expressions for the GTPC. Here, $\Pr(\,ON \mid i\,)$ is the probability that ACTIVE switch i is ON, and $\Pr(\,OFF \mid i\,)$ is the probability that the ACTIVE switch i is OFF. The probabilities for the INACTIVE states are all zero.

The entropy production, Equations S7, along with the normalization and free-energy constraints can be maximized with the method of Lagrange multipliers. Note that, in order to keep the value of the probabilities between zero and one, $k_i^{\pm}$ and $k_G^{\pm}$ cannot be smaller than J_i and J_G, respectively.

The total free-energy drop ΔG is finite. We quantify this by setting the average drop μ_0 over switches to be constant.

$$M\mu_0 \;=\; \sum_{i=1}^{N}\mu_i \;+\; \mu_G \;=\; \Delta G \tag{S10}$$

where μ_i and μ_G are the free-energy drops in the transit of PdPC and GTPC, respectively.

The entropy production rate with constraints in terms of Lagrange multipliers λ_1 and λ_2 is

$$T\,\sigma = -\sum_{i=1}^{N}[\,J_i\,\mu_i + \lambda_1\varphi_i + \lambda_2\gamma_i] - \lambda_1\varphi_G - \lambda_2\gamma_G \tag{S11}$$

$$\varphi_i = 1 \;-\; J_i\left(\frac{1}{k_i^-} + \frac{1}{k_i^+}\right) \tag{S12}$$

$$\varphi_G = 1 \;-\; \left(\sqrt{\frac{J_G}{q_G^-}} + \frac{J_G}{q_G^+}\right) \tag{S13}$$

$$\gamma_i = \mu_o - \mu_i \tag{S14}$$

$$\gamma_G = \mu_o - \mu_G \tag{S15}$$

The maximum production is obtained by taking the partial derivatives of Eq. S11 with respect to μ_i and J_i and then setting the derivative to zero.

The partial derivative with respect to μ_i and μ_G yields, for nonzero flux values,

$$0 \; = \; -J_i \; + \; \lambda_2 \tag{S16}$$

and

$$0 \; = \; -J_G \; + \; \lambda_2 \tag{S17}$$

Equations S16 and S17 can be written

$$J_i \; = \; J_G \; = \; J_0 \; = \; \frac{J}{M} \tag{S18}$$

where J is the total chemical flux into the system and J_0 is the average flux. The probabilities of a switch being ON and OFF is

$$\Pr(ON|\, i) = \frac{J_i}{k_i^-} \tag{S19}$$

and

$$\Pr(OFF|\, i) = \frac{J_i}{k_i^+} \tag{S20}$$

with comparable equations for G.

The partial derivative with respect to J_i and J_G yields, for nonzero values of flux,

$$0 \; = \; -\mu_i \; + \; \lambda_1 \left(\frac{1}{k_i^-} + \frac{1}{k_i^+} \right) \; \rightarrow \; -\mu_i + \frac{\lambda_1}{J_0} \tag{S21}$$

and

$$0 \; = \; -\mu_G \; + \; \lambda_1 \left(\frac{1}{2} \frac{1}{\sqrt{J_G \; k_G^-}} + \frac{1}{k_G^+} \right) \; \rightarrow \; -\mu_G \; + \; \frac{\lambda_1}{J_0} \left(1 - \frac{1}{2} \sqrt{\frac{J_0}{k_G^-}} \right) \tag{S22}$$

From Eq. S21 we see that the free-energy loss in each PdPC switch is constant

$$\mu_i = \mu_P = \text{constant} \tag{S23}$$

and

$$\mu_G \; = \; \mu_P \; \left(1 - \frac{1}{2} \sqrt{\frac{J_0}{k_G^-}} \right) \tag{S24}$$

The total free-energy drop is

$$M \; \mu_0 = M \; \mu_P \; - \frac{\mu_P}{2} \sqrt{\frac{J_0}{k_G^-}} \tag{S25}$$

which yields the expression for the free-energy drop in each ACTIVE PdPC.

$$\mu_P \; = \; \mu_0 \, \frac{M}{M - \sqrt{\frac{J_0}{k_G^-}}} \tag{S26}$$

0.2.2 Specialized BOIS Model

The receptors in the OFF state are pooled in the specialized model. In that case, mass conservation in the general model, Eqs. S8 and S9, is replaced with

$$J_i \;=\; \Pr(\,ON \mid i\,)\,k_i^- \;=\; C\;\Pr(\,OFF \mid i\,)\,k_i^+ \tag{S27}$$

$$J_G \;=\; \Pr(\,ON \mid G\,)^2\,q_G^- \;=\; C\;\Pr(\,OFF \mid G\,)\,q_G^+ \tag{S28}$$

where C is the receptor concentration of the C state, the part of the GPCR complex that excludes the ON states of the switches and the receptors unbound from ligands. The only difference between the specialized model and the general model is that Eq. S29 becomes

$$\Pr(OFF|\,i) \;=\; \frac{J_0}{C\,k_i^+} \tag{S29}$$

with a comparable expression for G. The specialization only affects the value of the forward reaction rate k_i^+ for ON and OFF states. The switch is OFF if

$$k_i^+ = \frac{J_0}{C} \tag{S30}$$

0.3 Switch Manipulation

Switch configurations can also change by interchange of switches. The first switch and the third switch in a configuration may swap locations on the C tail. If the one switch is ON and the other is OFF, then the configuration is altered by the exchange. Finally, the configuration can change by transmitting the information from one switch medium to another. For instance, a configuration may exist among the switches on the C tail of the GPCR complex. If the information is transmitted to the medium of effector coupling pathways, then the configuration will be altered. The same information will be transmitted, but the information will be described in a different way. The entire concept of switch location may be different in the two media.

The question remains as to what are the properties of the catalyst or catalysts that turn a switch ON and OFF. We have an example of a catalyst that does this. It has been observed that arrestin blockage of the G_α pathway in a GPCR complex is a situation in which a PdPC catalyzes a GTPC switch to turn OFF ((Violin et al., 2008, Eq. 7)). In the BOIS picture, this suggests that a phosphorylated site on the arrestin-bound C tail of the GPCR comes into contact with the GTPC, which is illustrated in Fig. S1B. The BOIS model predicts that switches must come in contact with each other in order to catalyze the switches to transition from ON to OFF and back. It is plausible that this can be achieved if the the C-tail/arrestin complex is flexible. It has been shown that arrestin-bound C tail is very flexible and can take on several conformations on and around the GPCR complex (Kahsai et al. (2018); Latorraca et al. (2018); Eichel et al. (2018)). In the GTPC case displayed in Fig. S1C, the ON state of a PdPC catalyzes the transition of the GTPC from ON to OFF by increasing the k_G^-. The GTPC OFF state inhibits k_2^- in a second PdPC, which turns that switch ON.

This suggests at least one mechanism for catalyzing the switches in general, illustrated in Fig. S1A. Here, a switch 1 in the ON state comes into contact with switch 3 in the ON state and catalyzes the transition of switch 3 from ON to OFF. The reaction takes k_3^-/J_0 from one to zero. The OFF state in switch 3 may also

inhibit the reaction represented by k_2^- to turn switch 2 OFF. In other words, an ON state of one switch can catalyze another switch to turn OFF, while the OFF state can inhibit another switch to turn ON.

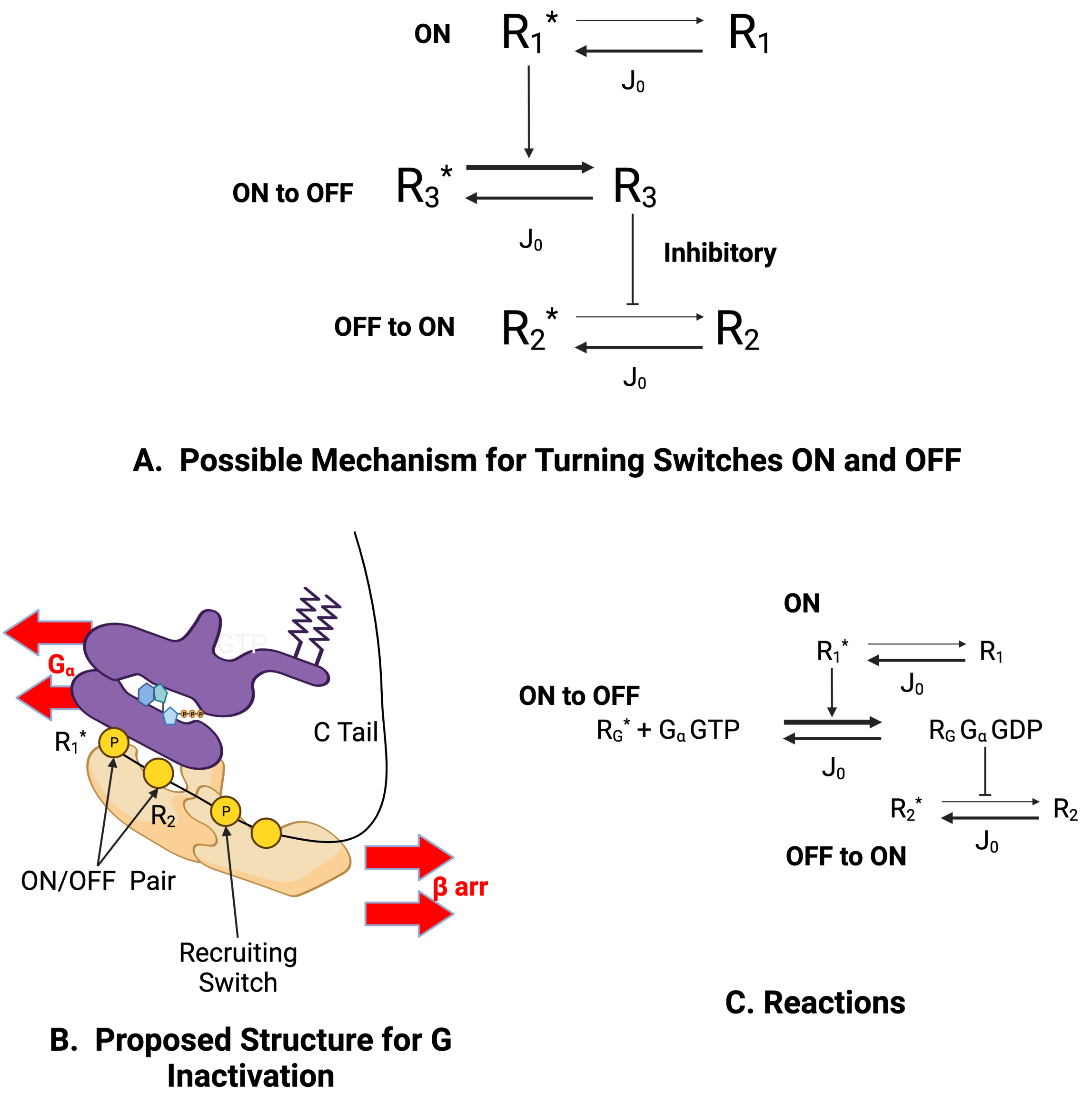

Figure S1. Speculative model: ON states on a switch catalyze another switch to go from OFF to ON, while OFF states of switch inhibit another switch's reaction rate k_i^- causing that switch to turn ON. **A.** Switch 1 comes within proximity to Switch 3. This is due to flexibility of C tail. The ON state of Switch 1 catalyzes Switch 3 to turn OFF (k_3^- becomes large). The OFF state of Switch 3 inhibits k_2^- and turns Switch 2 ON. **B.** Structure of the $G_\alpha GTP$ complex (purple) and the arrestin-bound C tail of the GPCR complex during inactivation of the G_α pathway. An initial configuration of phosphorylation switches represented by ON Recruiting Switch, recruits arrestin to the C tail of the GPCR complex. An ON phosphorylation site R_1^* on the arrestin/C-tail complex comes in contact with the G_α subunit turning the GTPC OFF. The OFF state of the GTPC inhibits the reaction from ON to OFF of an OFF phosphorylation switch R_2 to turn it ON, thus maintaining the number of ON switches and OFF switches. **C.** Chemical schematic in the form of **A.** to describe the inactivation of the G_α pathway.

These arguments have implications for transitions between and within the configuration groups illustrated in Fig. ?? for $M = 4$. If one switch catalyzes another switch to turn ON or OFF, then a transition is made between two groups in Fig. ??. If two switch states are changed as in Fig. S1A, then the switch configuration transitions between two configurations within a group. Changing the on-state of a single switch transitions between groups, while pairwise transition between switches within a group transition between two states within the group. Transitions within a group are particularly interesting. These transitions do not involve additional investment or loss of total information in the group as would occur if a sole switch were turned ON or OFF.

We propose here three mechanisms, $\mathcal{C}$, $\mathcal{P}$, and $\mathcal{T}$ for changing switch configurations while maintaining the number of ON switches. As mentioned, the first mechanism $\mathcal{C}$ changes of the switches by catalytically manipulating the return reaction rates k_i^- and k_G^- (Eqs. ?? and ??). We can represent this process mathematically as manipulation of a pair of ON/OFF states (0,1) and (1,0) and two pairs of states in which switches are both ON or both OFF (1,1) and (0,0).

$$\mathcal{C}(0,1) = (1,0) \tag{S31}$$

and

$$\mathcal{C}(1,0) = (0,1) \tag{S32}$$

Since the catalytic process of Fig. Fig. S1 only operates on two switches with opposite ON/OFF states, the $\mathcal{C}$ operator does nothing to the states (1,1) and (0,0), which implies

$$\mathcal{C}\mathcal{C} = \mathcal{I} \tag{S33}$$

where $\mathcal{I}$ is the identity operator. Two applications of $\mathcal{C}$ return you to your original state. In physics, this is known as charge symmetry (Lehnert (2016)).

The second mechanism $\mathcal{P}$ swaps the two switches from different sites. The transformed pair appears to be a mirror image of their pre-transformed configuration. This is a parity transformation. The parity operator $\mathcal{P}$ has the same set of transformations, Eqs. S31 and S35, as the catalytic operator $\mathcal{C}$. This implies, not only is the operator $\mathcal{P}\mathcal{P}$ invariant

$$\mathcal{P}\mathcal{P} = \mathcal{I} \tag{S34}$$

but also

$$\mathcal{P}\mathcal{C} = \mathcal{C}\mathcal{P} = \mathcal{I} \tag{S35}$$

the two operators $\mathcal{P}$ and $\mathcal{C}$ are $\mathcal{P}\mathcal{C}$ invariant (Lehnert (2016)).

The mechanisms $\mathcal{C}$ and $\mathcal{P}$ can both occur in the same population of switches, such as the switches on the C tail of the GPCR complex. The third mechanism $\mathcal{T}$ transitions between two distinct switch populations, such as the phosphorylation sites on the C tail and the conformational switches on the arrestin molecule (Latorraca et al. (2020)). In other words, the first transition in a cycle in Fig. ?? may occur on the C tail, while the next transition may occur as the information passes from the C tail to the arrestin molecule. The $\mathcal{T}$ transition can happen between any of the state pairs within a group (Fig. ??). A set of transitions is the identity operator if a cycle of transitions returns to its starting state.

$$(\mathcal{T})^n = \mathcal{I} \tag{S36}$$

where n is the total number of transitions $\mathcal{T}$ in the closed cycle. For $n = 2$, as would be the case if the chemical flow was from the C tail to the arrestin molecule and back, the cycle can be thought of as forward transition in time followed by a backward transition in time. This is known as time-reversal symmetry in physics (Lehnert (2016)). If we focus on the pair of switches, one with a location on the C tail and one with a location on the arrestin molecule that map into each other, then the mapping is equivalent to the $\mathcal{C}$ and $\mathcal{P}$ mappings:

$$\mathcal{T}(0,1) = (1,0) \tag{S37}$$

and

$$\mathcal{T}(1,0) = (0,1) \tag{S38}$$

if the switches change their ON/OFF state and the identity if they do not.

Therefore, any closed cycle of configurations within a group is invariant under some combination of the three operators $\mathcal{C}$, $\mathcal{P}$, and $\mathcal{T}$ This is known as $\mathcal{CPT}$ symmetry. What this means in non-mathematical language is that any state within a group can be accessed by a series of catalytic transitions between ON and OFF switch states, with an interchange of switch positions, and by transitioning from one switch medium to another (*eg.* C tail to arrestin). This transition can be of endogenous or pharmaceutical origins. Practically, this means that the three processes, turning switch pairs ON and OFF catalytically, swapping the locations of any two switches, and changing the locations of two switches on two separate switch media (C tail and arrestin) can change the code for effector coupling. Any state with the same number of ON states can be accessed by a combination of these processes. This increases the number of potential drug targets.

0.4 Simulation and Experiment

The MATLAB code that plots the figures is BOIS.mlx. Output inages are stored in the Graphs folder. The data files that the code reads are in the same folder. The Simbiology add-on must be installed to generate data files. The code for generating the BOIS data is in Simbiology project Central.sbproj. The code for generating Mass-Action data is in MassAction.sbproj.

0.4.1 Dose Response

We examine the relationship between forward and backward processes in the constrained BOIS model of Fig. S2B, which is used in the simulation. The flux relationship for ON switches, $(R_i^* = 1)$, is

$$J_0 = Ck^+ = k^- \quad \text{ON switches} \tag{S39}$$

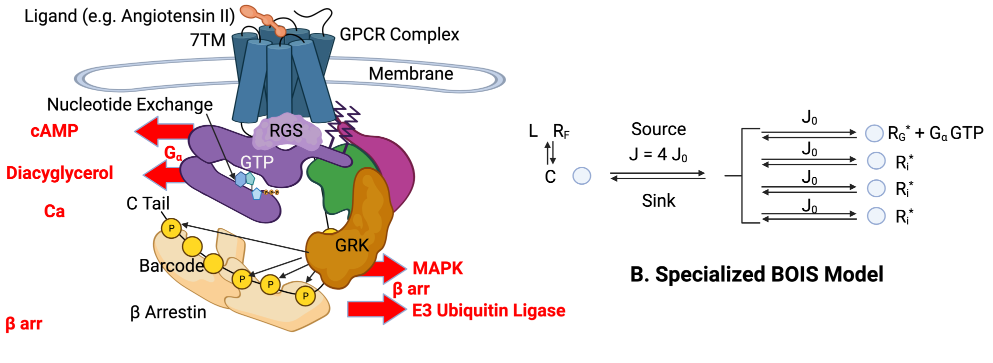

A. Adrenergic and Angiotensin

B. Specialized BOIS Model

Figure S2.

The total normalized receptor concentration R_T is

$$R_T = C + M_o + R_F \tag{S40}$$

where R_F is the normalized concentration of free receptors, those not bound to a ligand.

$$R_F = \frac{K_D}{L + K_D} \left(R_T - M_o\right) \theta(R_T - M_o) \quad \text{ON switches} \tag{S41}$$

where θ is the Heaviside step function, L is the ligand concentration and K_D is the ligand-receptor dissociation constant. The step function is required to keep the concentration R_F equal to or greater than zero. The ligand-bound concentration C is

$$C = \left(\frac{L}{L + K_D}\right) \left(R_T - M_o\right) \theta(R_T - M_o) \tag{S42}$$

A set of numerical experiments were performed to determine the threshold for activation of the switches. The architecture was composed of four switches, one GTPC and three PdPCs; of which one, two, three, or four were ON, while the remaining were OFF. All receptors were initially in state C. No receptors were initially in the ligand-free state or in the switch ON states. The total number of ON switches was $M_o = (1, 2, 3, 4)$. We found that switches did not activate ($J_0 > 0$) unless $C > M_o$ at the initial time $t = 0$. Once an ON ($k_i^- = J_0$) switch was activated, it remained activated irrespective of the concentration C. For cases in which the normalized concentration C did not initially exceed the number of ON switches M_o, the simulator failed to evolve to a computable steady-state solution. This indicates that physically meaningful numerical solutions are not possible if the normalized receptor concentration in state C is less than M_o during an initial increase in ligand concentration. In the initial sub-threshold situation, the concentration C may be nonphysically driven below zero, or the code is simply unable to find an integration time step for any finite value of J_0. Once the ligand concentration exceeds the value in which C is greater than M_o, then $2M_o$ switches are activated with half the switches in the ON state and half in the OFF state. If the concentration C subsequently drops below M_o, the ACTIVATED switches remain activated. No nonphysical values of concentration are generated and the integration runs smoothly.

In a second set of numerical experiments that mimicked assay experiments, receptors were initialized to lie in the ligand-free state R_F instead of the ligand-bound OFF state C. The ligand concentration starts at zero and slowly increases to a value much larger than the dissociation constant K_D. As the ligand concentration increases, receptors move from state R_F to the GPCR-complex state C. At low values of C concentration, the number of receptors in the ON state of the switches is zero, no flux is permitted into the switch. When the complex receptor concentration C exceeds the ON switch number M_o, the flux J_0 turns on for both ON and OFF switches. Once this event has occurred, the switches remains active even if the concentration C drops below the activation threshold. The activation threshold is given by

$$C \;=\; \left(\frac{L}{L+K_D}\right)\; R_T \;\geq\; M_o \tag{S43}$$

We define EC_{50} as the ligand concentration L at which the equality holds.

$$EC_{50} = \frac{M_o\; K_D}{R_T - M_o} \tag{S44}$$

Runs with the ligand concentration normalized to EC_{50} and for values of ON switch number $M_o = (1, 2)$ and for total normalized receptor concentration $R_T = (0.1, 1, 10, 100)$ are displayed in Fig. S3A. The figure illustrates dose-response curves for both PdPCs and GTPCs. Note that the curves are nearly universal when displayed as a function of the ratio of ligand concentration L to EC_{50}. This implies that the dose-response curves are completely characterized by the quantity EC_{50} defined in Eq. S56.

0.4.2 Differing Responses for Rigid and Flexible Proteins

Equation S56 can be interpreted in two ways depending on whether the receptor proteins are rigid or flexible. For rigid proteins, the number of ON switches is a constant and determined by the rigid bonds among protein elements. In that case, the ON number M_o and the total receptor concentration R_T determine the single common ligand concentration EC_{50} to which all the dose-response curves are scaled. Equation S56 indicates that for rigid proteins in which the number of ON switches is constant for the ensemble, the ON switches become ACTIVATED at a common value of $L = EC_{50}$. In other words, all switches have the same value for EC_{50}. This is not seen in experimental assays Rajagopal et al. (2011). Rather, different downstream pathways are associated with different values of EC_{50}. This is further evidence that switches cannot be completely composed of rigid proteins.

The number of ON switches M_o available for activation can be mutable in flexible proteins, however. The protein environment, including the ligand binding, may select the number of ON switches available to be activated at any given value of ligand concentration L. In that case, Eq. S56 can be interpreted as determining the number ON switches M_o in the complex given the total receptor concentration R_T and EC_{50}. Moreover, it is well known Lefkowitz et al. (1993) that the activation of G proteins is a tertiary reaction among the ligand, receptor, and G protein. This implies that the downstream pathways affect the ligand dissociation constant K_D. We allow for the possibility that the PdPC activation can also affect the ligand dissociation K_D.

In the case of flexible protein conformations, the picture that emerges is:

1. At the initial time $t = 0$, the ligand concentration L is zero as are the GPCR -complex concentration C. The number of ON switches is zero as is the number of OFF switches.
2. The ligand concentration L is increased until it reaches a point in which Eq. S56 for $M_o = 1$ is satisfied.

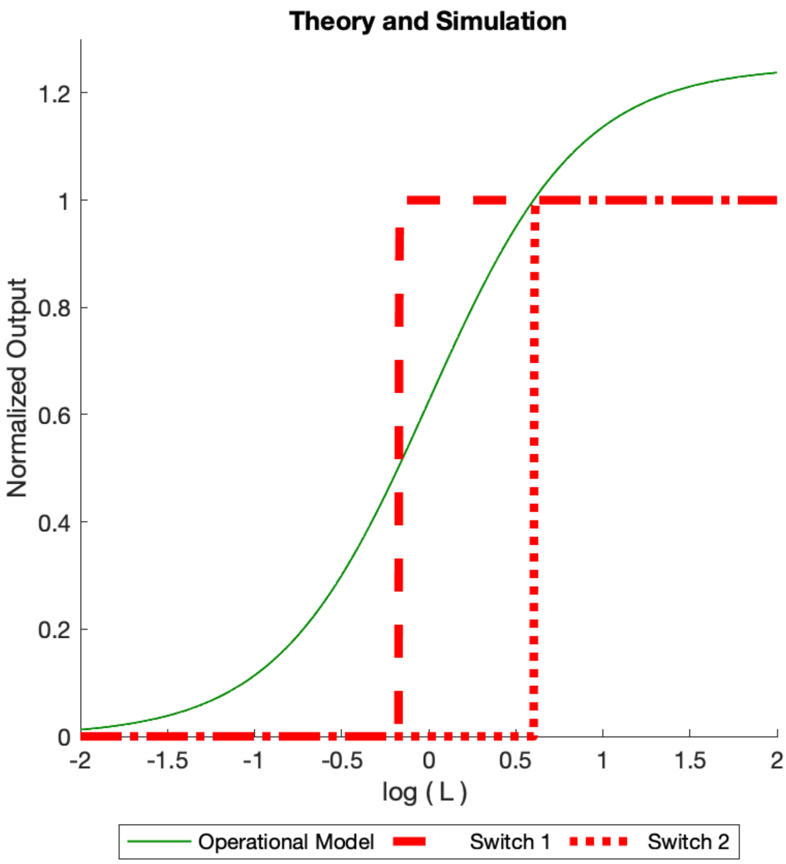

Figure S3. **A. Dose Response** Simulated (BOIS) dose response curves where $EC_{50} = M_o K_D/(R_T - M_o)$ for two values of $M_o = (1, 2)$ and four values of $R_T = (0.1, 1, 10, 100)$. The dose response curves are nearly universal for this scaling. The curves can be characterized by the reference concentration R_{ref} and the EC_{50}. **B. Curves of constant total receptor concentration.** The total receptor concentration varies from $R_T = 1.5$ to $R_T = 10.5$ in steps of 1 in normalized concentration units. The curve for $R_T = 1.5$ is the single point on the left. The curve for $R_T = 10.5$ is the curve containing 10 points on the right. We see that increasing the number of active switches $M = 2M_o$ while holding the receptor concentration constant increases EC_{50} for ligand-receptor complex.

3. At that point an ON ($k_i^- \to J_0$) switch is ACTIVATED ($J_0 > 0$) while an OFF ($k_i^- \to \infty$) switch is ACTIVATED, thus satisfying the requirement that the number of ACTIVE ON switches is equal to the number of ACTIVE OFF switches.

4. The concentration of state C is drained into the new ACTIVE ON switch and the process repeats.

5. Switches are ACTIVATED as the ligand concentration is increased until the addition of a new ON switch would require more receptors than are available at the given total receptor concentration R_T.

We can make this argument quantitative. We define M_o^{max} as the maximum possible number of ON switches M_o, both ACTIVE and INACTIVE.

$$R_T = M_o^{max} + \Delta R_T \tag{S45}$$

where the excess receptor concentration ΔR_T is

$$0 < \Delta R_T \leq 1 \tag{S46}$$

Initial values are $L = 0$, $M_o = 0$, $R_F = R_T$, and $C = 0$. The ligand concentration L increases until it reaches a value in which the threshold requirement, Eq. S43, is reached for $M_o = 1$.

$$C \;=\; 1 \;=\; \left(\frac{L}{L + K_D}\right)\; R_T \;=\; \left(\frac{EC_{50}(1)/K_D(1)}{EC_{50}(1)/K_D(1) + 1}\right)\; (M_o^{max} + \Delta R_T) \tag{S47}$$

where the arguments of EC_{50} and K_D indicate that this is the first threshold that is met. When this ligand-concentration threshold is met, the receptors drain into the newly ACTIVATED ON state and the GPCR-complex concentration C becomes

$$C \;\rightarrow\; \left(\frac{L}{L + K_D}\right)\; (R_T \;-\; 1) \;=\; \left(\frac{L}{L + K_D}\right)\; (M_o^{max} + \Delta R_T \;-\; 1) \tag{S48}$$

The first estimate for $R_T = M_o^{max} + \Delta R_T$ is

$$(M_o^{max} +\; \Delta R_T)_1 \;\approx\; \frac{EC_{50}(1)/K_D(1) + 1}{EC_{50}(1)/K_D(1)} \tag{S49}$$

The ligand concentration L continues to increase until the second threshold is reached and another pair of switches become activated.

$$C \;=\; 1 \;=\; \left(\frac{L}{L + K_D}\right)\; (R_T - 1) \;=\; \left(\frac{EC_{50}(2)/K_D(2)}{EC_{50}(2)/K_D(2) + 1}\right)\; (M_o^{max} + \Delta R_T - 1) \tag{S50}$$

The second measurement of R_T is

$$(M_o^{max} +\; \Delta R_T)_2 \;\approx\; 1 \;+\; \frac{EC_{50}(2)/K_D(2) + 1}{EC_{50}(2)/K_D(2)} \tag{S51}$$

The next estimate is

$$(M_o^{max} +\; \Delta R_T)_3 \;\approx\; 2 \;+\; \frac{EC_{50}(3)/K_D(3) + 1}{EC_{50}(3)/K_D(3)} \tag{S52}$$

In general, the nth estimate is

$$(M_o^{max} +\; \Delta R_T)_n \;\approx\; (n - 1) \;+\; \frac{EC_{50}(n)/K_D(n) + 1}{EC_{50}(n)/K_D(n)} \tag{S53}$$

The process continues until the final estimate of R_T is

$$(M_o^{max} +\; \Delta R_T)_{final} \;\approx\; (M_o^{max} - 1) \;+\; \frac{EC_{50}(M_o^{max})/K_D(M_o^{max}) + 1}{EC_{50}(M_o^{max})/K_D(M_o^{max})} \tag{S54}$$

or

$$\Delta R_T|_{final} \;\approx\; \frac{EC_{50}(M_o^{max})/K_D(M_o^{max}) + 1}{EC_{50}(M_o^{max})/K_D(M_o^{max})} \;-\; 1 \tag{S55}$$

The flexible version of Eq. S43 is, for the nth vale of EC_{50}/K_D,

$$EC_{50}(n) = \frac{K_D(n)}{R_T - n} \quad \text{Flexible} \tag{S56}$$

where $1 \leq n \leq M_o^{max}$. This is displayed as the black curves in Fig.S3B. The comparable expression for Rigid proteins is, from Eq. S56,

$$EC_{50}(M_o) = \frac{M_o \, K_D(M_o)}{R_T - M_o} \quad \text{Rigid} \tag{S57}$$

This is displayed as red curves in Fig. S3B.

0.4.3 Observed Dissociation

Each pathway has its distinct ligand dissociation value EC_{50}. We can also calculate a global dissociation K_{GD} constant defined as the ligand value at which half of the receptors are free of ligands and half are bound with ligands. This is quite different from the EC_{50} value calculated for a given downstream response to a given ligand. In that case, the dissociation EC_{50} is the ligand concentration at which a switch becomes activated. Intuitively, it can be thought of as the ligand concentration at which a switch is halfway turned ON, at which the receptor concentration in the ON state is half the reference concentration.

This dissociation is also quite different from the dissociation constant K_D determined by the reaction rates in ligand/receptor binding. Here, K_D is associated with the reaction

$$\mathrm{R_F} + \mathrm{L} \underset{\mathrm{k^-}}{\overset{\mathrm{k^+}}{\rightleftharpoons}} \mathrm{C} \tag{S58}$$

where the concentration of C, denoted by C, is related to the free-receptor concentration R_F and the total receptor concentration R_T by

$$C = R_T - R_F \tag{S59}$$

This is the case in which no switches are ACTIVATED. In that case,

$$K_D = \frac{k^-}{k^+} \tag{S60}$$

which is quite different from the the dissociation constants when switches are ACTIVATED. It seems to be very important to identify in experiments, which type of dissociation is being observed.

To calculate K_{GD}, note that the concentration of ligand-free receptors is

$$R_F \;=\; \left(\frac{1}{L + K_D}\right) \, (R_T - M_o) \tag{S61}$$

The observed value of the dissociation constant K_D^{obs} occurs when the ligand-free concentration is half the total concentration

$$\frac{R_T}{2} \;=\; \left(\frac{1}{K_{GD} + K_D}\right) \, [R_T - M_o(1/2)] \tag{S62}$$

where $M_o(1/2)$is the value of ON switch number that corresponds to half the total number of switches being ON. If M_o is even, then

$$M_o(1/2) = \frac{M_o^{max}}{2} \tag{S63}$$

If M_o is odd then

$$M_o(1/2) = \frac{M_o^{max} - 1}{2} \tag{S64}$$

Equation S63 can be written

$$R_T \;=\; M_o^{max} + \Delta R_T \;=\; 2\; M_o(1/2)\; \left(\frac{K_D}{K_D - K_{GD}}\right) \tag{S65}$$

$$\Delta R_T \;=\; 2\; M_o(1/2)\; \left(\frac{K_D}{K_D - K_{GD}}\right) \;-\; M_o^{max} \tag{S66}$$

For even values of M_o^{max}

$$0 \;\leq\; \Delta R_T \;=\; M_o^{max}\; \frac{K_{GD}}{K_D - K_{GD}} \;\leq\; 1 \tag{S67}$$

For odd values

$$0 \;\leq\; \Delta R_T \;=\; M_o^{max}\; \frac{K_{GD}}{K_D - K_{GD}} \;-\; \frac{K_D}{K_D - K_{GD}} \;\leq\; 1 \tag{S68}$$

This puts a constraint of the actual ligand dissociation constant K_D and on the maximum number of ON switches M_o^{max}. For even values of M_o^{max}, the largest possible value for M_o^{max} is given by

$$M_o^{max} \;\leq\; \frac{K_D - K_{GD}}{K_{GD}} \quad \text{Even} \tag{S69}$$

For odd values

$$M_o^{max} \;\leq\; \frac{2\; K_D - K_{GD}}{K_{GD}} \quad \text{Odd} \tag{S70}$$

0.4.4 Experimental Observations

Observations of EC_{50} from Ref. Rajagopal et al. (2011) are given in Table S1.

Assay results on two different receptors, Adrenergic and Angiotensin II, are displayed. Two different downstream pathway concentrations were measured for each receptor, the G_α pathway and one β-arr pathway. Other pathways may have been present, but they were not measured. The estimated reference receptor concentration R_{ref} was taken to be the maximum Formoterol concentration for the Adrenergic receptor and Angiotensin II maximum concentration for the Angiotensin II receptor. It should be noted that the experimental observation used a common reference concentration for receptor switch states before its theoretical prediction by the BOIS model. The maximum normalized downstream concentrations R_{max} for each pathway are given in columns marked G_α and β arr. The EC_{50} concentrations are measured in moles per liter. Several distinct ligands, listed in the table, were applied to each receptor.

REFERENCES

Berg Jeremy, M., Tymoczko John, L., Gatto Jr Gregory, J., and Lubert, S. (2019). *Biochemistry* (W. H. Freeman)

Table S1. Assay Data. Assay values for maximum stimulus for Adrenergic and Angiotensin receptors Rajagopal et al. (2011). The concentrations G_α and β arr are normalized to the reference value R_{ref}. Ligand concentrations are molar. The ligand names correspond to the names used in Ref. Rajagopal et al. (2011). Balanced ligands for the Adrenergic receptor are Formoterol, Isoproterenol, Epinephrine, and Fenoterol. Balance ligands for the Angiotensin II receptor are Angiotensin II, TRV0120055, TRV0120056, A1, and S1C4. Adrenergic ligands that are G_α biased are Dobutamine, Norepinephrine, Clenbuterol, Salmeterol, and Salbutamol. No adrenergic ligands in the sample are β-arr biased. Angiotensin II ligands that are β-arr biased are TRV0120044, TRV0120045, TRV0120034, and S1G4G8. No angiotensin ligands in the sample are G_α- biased.

**Adrenergic**					**Angiotensin II**				
	G_α	$\log(EC_{50})$	β Arr	$\log(EC_{50})$		G_α	$\log(EC_{50})$	β Arr	$\log(EC_{50})$
Form	1.05	-9.62	1.02	-8.61	TRV120056	0.95	-7.34	1.00	-6.42
Iso	0.87	-9.71	0.94	-8.14	TRV120055	1.00	-7.97	1.03	-7.05
Fen	0.80	-9.41	0.94	-7.81	AngII	1.00	-8.84	1.00	-7.90
Epi	0.88	-9.01	0.69	-7.25	S1C4	0.93	-7.63	0.70	-6.66
Salb	0.92	-8.30	0.33	X	A1	0.98	-8.52	0.95	-7.68
Salm	0.97	-8.26	0.33	X	TRV120034	0.12	X	0.89	-7.63
Clen	1.00	-8.85	0.17	X	TRV120026	0.09	X	0.89	-6.64
Norepi	0.96	-6.84	0.21	X	TRV120045	0.11	X	0.89	-7.57
Dob	0.94	-6.57	0.04	X	SGG	0.17	X	0.72	-5.69
Pind	0.10	X	0.01	X	TRV120044	0.09	X	0.76	-6.79

Eichel, K., Jullié, D., Barsi-Rhyne, B., Latorraca, N. R., Masureel, M., Sibarita, J.-B., et al. (2018). Catalytic activation of β-arrestin by gpcrs. *Nature* 557, 381–386

Kahsai, A. W., Pani, B., and Lefkowitz, R. J. (2018). Gpcr signaling: conformational activation of arrestins. *Cell Research* 28, 783–784

Latorraca, N. R., Masureel, M., Hollingsworth, S. A., Heydenreich, F. M., Suomivuori, C.-M., Brinton, C., et al. (2020). How gpcr phosphorylation patterns orchestrate arrestin-mediated signaling. *Cell* 183, 1813–1825

Latorraca, N. R., Wang, J. K., Bauer, B., Townshend, R. J., Hollingsworth, S. A., Olivieri, J. E., et al. (2018). Molecular mechanism of gpcr-mediated arrestin activation. *Nature* 557, 452–456

Lefkowitz, R. J., Cotecchia, S., Samama, P., and Costa, T. (1993). Constitutive activity of receptors coupled to guanine nucleotide regulatory proteins. *Trends in pharmacological sciences* 14, 303–307

Lehnert, R. (2016). Cpt symmetry and its violation. *Symmetry* 8, 114

Qian, H. (2003). Thermodynamic and kinetic analysis of sensitivity amplification in biological signal transduction. *Biophysical chemistry* 105, 585–593

Qian, H. (2007). Phosphorylation energy hypothesis: open chemical systems and their biological functions. *Annu. Rev. Phys. Chem.* 58, 113–142

Qian, H. and Reluga, T. C. (2005). Nonequilibrium thermodynamics and nonlinear kinetics in a cellular signaling switch. *Physical review letters* 94, 028101

Qian, H. and Roy, S. (2012). An information theoretical analysis of kinase activated phosphorylation dephosphorylation cycle. *IEEE Transactions on NanoBioscience* 11, 289–295

Rajagopal, S., Ahn, S., Rominger, D. H., Gowen-MacDonald, W., Lam, C. M., DeWire, S. M., et al. (2011). Quantifying ligand bias at seven-transmembrane receptors. *Molecular pharmacology* 80, 367–377

Selimkhanov, J., Taylor, B., Yao, J., Pilko, A., Albeck, J., Hoffmann, A., et al. (2014). Accurate information transmission through dynamic biochemical signaling networks. *Science* 346, 1370–1373

Violin, J. D., DiPilato, L. M., Yildirim, N., Elston, T. C., Zhang, J., and Lefkowitz, R. J. (2008). β2-adrenergic receptor signaling and desensitization elucidated by quantitative modeling of real time camp dynamics. *Journal of biological chemistry* 283, 2949–2961
